# Efficacy and safety of *Prunella vulgaris* L. combined with antithyroid drugs for hyperthyroidism: a systematic review and meta-analysis

**DOI:** 10.3389/fphar.2025.1530152

**Published:** 2025-02-27

**Authors:** Maoying Wei, Qiyao Zhao, Mingyi Yuan, Yuyun Fan, Mingdi Li

**Affiliations:** ^1^ Dongzhimen Hospital, Beijing University of Chinese Medicine, Beijing, China; ^2^ Guang’anmen Hospital, China Academy of Chinese Medical Sciences, Beijing, China; ^3^ Department of Traditional Chinese Internal Medicine, Linyi People’s Hospital, Linyi, China

**Keywords:** hyperthyroidism, *Prunella vulgaris* L., antithyroid drugs, systematic review, meta-analysis

## Abstract

**Objective:**

To systematically evaluate the efficacy and safety of *Prunella vulgaris* L. (PVL) preparations combined with antithyroid drugs (ATDs) for the treatment of hyperthyroidism.

**Methods:**

Eight Chinese and English databases were searched for randomized controlled trials (RCTs) comparing PVL preparations combined with ATDs and ATDs for hyperthyroidism treatment. The Cochrane risk-of-bias assessment tool was used to evaluate the quality of included studies. Statistical analyses were performed using the Revman 5.3 software. Stata software (version 16.0) was used to detect publication bias. The GRADE system was used to assess the level of evidence.

**Results:**

Seventeen studies were analyzed. The total sample size was 1,366 patients. Meta-analysis revealed that treatment with PVL preparations in combination with ATDs effectively reduced free triiodothyronine [standardized mean difference (SMD) = −0.98, 95%CI (−1.39, −0.57), *P* < 0.00001], free thyroxine [SMD = −0.82, 95% confidence interval (CI) (−1.16, −0.47), *P* < 0.00001], thyrotropin receptor antibody [SMD = −1.11, 95%CI (−1.52, −0.71), *P* < 0.00001], thyroid isthmus thickness [mean difference (MD) = −0.13, 95%CI (−0.15, −0.10), *P* < 0.00001], width of left thyroid lobe [MD = −0.22, 95%CI (−0.27, −0.17), *P* < 0.00001], thickness of left thyroid lobe [MD = - 0.22, 95%CI (−0.33, −0.10), *P* = 0.0003], length of left thyroid lobe [MD = −0.63, 95%CI (−0.79, −0.47), *P* < 0.00001], width of right thyroid lobe [MD = −0.21, 95%CI (−0.26, −0.16), *P* < 0.00001], thickness of right thyroid lobe [MD = −0.27, 95%CI (−0.32, −0.22), *P* < 0.00001], length of right thyroid lobe [MD = −0.45, 95%CI (−0.61, −0.28), *P* < 0.00001], incidence of adverse events [risk ratio (RR) = 0.34, 95%CI (0.24, 0.50), *P* < 0.00001], tumor necrosis factor-α [SMD = −2.05, 95%CI (−2.85, −1.25), *P* < 0.00001], and increasing thyroid-stimulating hormone [SMD = 0.71, 95%CI (0.43, 0.99), *P* < 0.00001], and interleukin-10 [MD = 1.73, 95%CI (1.35, 2.10), *P* < 0.00001] better than that of ATDs alone. Combination therapy with PVL preparations was comparable to the efficacy of ATDs alone in improving relapse rates and interleukin-6 and interferon gamma levels.

**Conclusion:**

Treatment of hyperthyroidism with PVL preparations in combination with ATDs was superior to treatment with ATDs alone in terms of improvements in thyroid function, thyroid antibodies, thyroid gland size, inflammation, and incidence of adverse events. However, owing to the low strength of evidence from the included studies, this conclusion requires further validation in more high-quality RCTs.

**Systematic Review Registration:**

https://www.crd.york.ac.uk/prospero, identifier CRD42024572591.

## Introduction

Hyperthyroidism is an endocrine disorder caused by inappropriate and sustained synthesis and secretion of excess thyroid hormones by the thyroid gland. Clinical manifestations are primarily caused by excess circulating thyroid hormones, and the severity is related to factors such as the length of history, degree of hormone elevation, and age. Common symptoms include a fear of heat, excessive sweating, excessive appetite, weight loss, fatigue, palpitations, anxiety, irritability, and insomnia. Hyperthyroidism affects 0.2%–1.3% of the global population (Wiersinga et al., 2023). The most common cause of hyperthyroidism is Graves’ disease (GD) or toxic nodular goiter. A study in 31 provinces in China reported prevalence rates of 0.78% for overt hyperthyroidism, 0.44% for subclinical hyperthyroidism, and 0.53% for GD (Li et al., 2020b). Overt hyperthyroidism and GD are most common in women (overt hyperthyroidism: 1.16% VS 0.64%, *P* < 0.001; GD: 0.65% VS 0.37%, *P* < 0.001) (Wang et al., 2021). The peak prevalence is observed between 30 and 60 years of age, with a significant decrease in prevalence after 60 years of age (Wang et al., 2021).

Almost all the cells in the body are affected by thyroid hormones. If left untreated, hyperthyroidism can lead to an increased risk of atrial fibrillation, osteoporosis, and death (Lee and Pearce, 2023; Kostopoulos and Effraimidis, 2024). A cross-sectional study suggested that hyperthyroidism significantly increased the risk of osteoarthritis [odds ratio (OR) = 2.23, 95% confidence interval (CI) = 1.2–4.17] (Zhao et al., 2024). Bode et al. (2022) found that patients have a higher risk of clinical depression than those with normal thyroid function (OR = 1.67, 95% CI = 1.49–1.87). Antithyroid drugs (ATDs), radioiodine therapy, and surgery are the current treatment options for hyperthyroidism. ATDs are the first-line treatment for GD. However, ATDs have the disadvantages of a long treatment period, susceptibility to adverse reactions during treatment (e.g., impaired liver function, peripheral blood leukopenia, and allergic rashes), and susceptibility to relapse after drug discontinuation.

Chinese herbal medicines are effective in the treatment of hyperthyroidism (Ma et al., 2023; Yang et al., 2024). In 2010, Chang and Huang (2010) reported on a 33-year-old woman with GD who developed urticaria and itchy skin after taking ATDs and sought traditional Chinese medicine treatment. After 3 years of treatment with Jia Wei Xiao Yao San plus *Prunella vulgaris* L. (PVL, also known as Xiakucao), *Fritillaria thunbergii* Miq. (Liliaceae; Fritillariae thunbergii bulbus), and *Ostrea gigas* Thunberg (Ostreidae; Ostreae concha), her symptoms subsided, and her thyroid function returned to normal. Similarly, Lin et al. (2021) reported that Jia Wei Xiao Yao San, PVL, *Fritillaria thunbergii* Miq. (Liliaceae; Fritillariae thunbergii bulbus), and *Ostrea gigas* Thunberg (Ostreidae; Ostreae concha) are safe and effective formulations for the treatment of hyperthyroidism. PVL was first recorded in Shen Nong’s Classic of Materia Medica (Shen Nong Ben Cao Jing). It is the dried fruit spike of PVL of the Labiatae family. It is nominated because of its blooming characteristics in spring and withering after summer. PVL is bitter, pungent, and cold, and enters the liver and gall bladder meridians, according to the 2020 edition of the Chinese Pharmacopoeia. It clears and purges the liver fire, brightens the eyes, dissipates nodulation, and reduces swelling. Modern pharmacological studies have shown that PVL has various biological activities, including antioxidant, anti-inflammatory, immunomodulatory, antitumor, antiviral, antibacterial, antihypertensive, hypoglycemic, hypolipidemic, and hepatoprotective activities (Pan et al., 2022). As a clinically used botanical drug, PVL preparations (PVL capsules, PVL granules, PVL tablets, PVL oral solution, etc.) are recommended in China for the treatment of a variety of thyroid disorders, such as GD, goiter, nodular thyroid disease, and Hashimoto’s thyroiditis (Standardization Project Team for the Guidelines on Clinical Application of Chinese Patent Medicine for the Treatment of Dominant Diseases, 2022; Experts of Clinical Application of Xiakucao Oral Liquid, 2020). The addition of PVL preparations to ATD therapy improves clinical efficacy, reduces adverse events, and decreases relapse rates in patients with hyperthyroidism (Kan and Yi, 2020; Chai et al., 2020; Yue and Shi, 2020). However, these studies were single-center, small-sample clinical trials. In this study, we systematically evaluated the efficacy and safety of PVL preparations combined with ATDs for the treatment of hyperthyroidism to provide evidence for their clinical application.

## Methods

This study was performed in accordance with the Cochrane Handbook for Systematic Reviews (Higgins et al., 2021) and the guidelines of the Preferred Reporting Items for Systematic Reviews and Meta-Analyses (PRISMA) (Page et al., 2021). The details are presented in Supplementary Table S1. The study protocol was registered with the International Prospective Register of Systematic Reviews (PROSPERO). The registration number was CRD42021297266.

### Eligibility criteria

(1) Types of studies: randomized controlled trials (RCTs).
(2) Participants: Clinical Diagnosis of Hyperthyroidism. No restrictions were imposed on the age, sex, disease duration, region, or race. The diagnostic criteria were based on the guidelines for the Diagnosis and Treatment of Thyroid Diseases in China (2007) (Writing Group of Chinese Thyroid Disease Diagnosis and Treatment Guidelines, 2007), the guidelines for Primary Care of Hyperthyroidism (2019) (Chinese Society of General Practice, Editorial Committee of the Chinese Journal of General Practitioners of the Chinese Medical Association, 2019) and related books.
(3) Types of Intervention: The control group was treated with oral ATDs (methimazole, carbimazole, or propylthiouracil). The experimental group was orally administered the PVL preparations in combination with ATDs. There were no limits to the dosage form, duration of treatment, or daily dosage of the PVL preparations.
(4) Types of outcomes: Primary outcomes were free triiodothyronine (FT3), free thyroxine (FT4), and thyroid-stimulating hormone (TSH). Secondary outcomes were thyrotropin receptor antibody (TRAb), thyroid gland size, tumor necrosis factor-α (TNF-α), interleukin-6 (IL-6), interleukin-10 (IL-10), interferon gamma (IFN-γ), relapse rate, and adverse events.

### Exclusion criteria

(1) Animal experiments, case reports, retrospective studies, conference abstracts, reviews, meta-analyses, etc.; (2) Literature for which it was not possible to access the full text or extract valid data. (3) Duplicated studies (4) Experimental group used botanical drugs other than the PVL preparations. (5) There have been no previous studies on these outcome indicators.

### Search strategies

Computerized searches were performed on eight major Chinese and English databases: PubMed, Cochrane Library, Embase, Web of Science, Wanfang Data, China National Knowledge Infrastructure (CNKI), Chongqing Chinese Science and Technology Journal Database (VIP), and China BioMedical Literature Service System (CBM) to collect RCTs on PVL preparations combined with ATDs for the treatment of hyperthyroidism. The search period was from the establishment of each database until 18 July 2024. The search was conducted using a combination of subject terms and free words. The key search terms include “Hyperthyroidism, or Hyperthyroid, or Graves’ disease,” and “Prunella vulgaris, or Prunella, or Xiakucao.” Additionally, we manually searched the references of the included studies to obtain additional relevant studies. The details of the search are presented in Supplementary Table S2.

### Study selection and data extraction

After eliminating duplicates using NoteExpress V3.4.0.8878 document management software, the literature was initially screened by reading titles and abstracts according to the inclusion and exclusion criteria. After excluding studies that did not meet the inclusion criteria, the remaining literature was read in its entirety to determine the final inclusion. Literature screening and data extraction were performed independently by two researchers and crosschecked at the end of each step. In cases of disagreement, a decision was made through discussion or after conferring with a third researcher. An Excel spreadsheet was used to extract the feature information of the included studies. The extraction included (1) basic information about the study, such as the first author, year of publication, and sample size; (2) basic information about the study participants, such as age, sex, disease duration, and diagnostic criteria; (3) interventions in the experimental and control groups; (4) outcomes; (5) methodological quality assessment entries. If an outcome was measured at more than one time point, data from the longest time point were included.

### Assessment of risk of bias

The included RCTs were evaluated for risk of bias by two researchers using the Cochrane risk-of-bias assessment tool. If there were discrepancies, a decision was made through discussion or after conferring with a third researcher. Entries for the methodological quality evaluation included random sequence generation, allocation concealment, blinding of participants and personnel, blinding of outcome assessments, incomplete outcome data, selective reporting, and other biases. Each item was categorized as “low risk,” “unclear,” or “high risk” according to the level of risk of bias.

### Evidence quality evaluation

The Grading of Recommendations Assessment, Development, and Evaluation (GRADE) system was used to grade the quality of evidence for the outcomes of the included studies. The evaluation included five aspects: study limitations, inconsistencies, indirectness, imprecision, and publication bias.

### Data synthesis and statistical analysis

Statistical analyses were performed using the Revman 5.3 software. For dichotomous variables, the risk ratio (RR) was used as an effect size. Continuous variables were defined as the mean difference (MD) or standardized mean difference (SMD) as effect sizes. A 95% confidence interval (CI) was calculated for each effect size. When the data reported in the included studies were presented as medians and quartiles, the mean and standard deviation were estimated by referring to the relevant literature (Luo et al., 2018; Wan et al., 2014). Heterogeneity between the included studies was analyzed using Cochran’s Q test and combined with I^2^ to quantify the magnitude of heterogeneity. According to the Cochrane Handbook, I^2^ ≥ 25% is considered mild heterogeneity, I^2^ ≥ 50% is considered moderate heterogeneity, and I^2^ ≥ 75% is considered severe heterogeneity. If the heterogeneity among the studies was small (*P* > 0.1, I^2^ ≤ 50%), a meta-analysis was performed using a fixed-effects model. Conversely (*P* < 0.1, I^2^ > 50%), a meta-analysis was performed using a random-effects model. For outcomes with significant heterogeneity (I^2^ ≥ 50%), this study plans to conduct subgroup analysis for type of PVL preparations, duration of treatment, and sample size to explore the sources of heterogeneity. A sensitivity analysis was conducted by omitting individual studies to assess the robustness of the synthesized results. When there were no fewer than 10 included studies for an outcome indicator, the presence of publication bias was analyzed using Egger’s test (Stata 16.0 software). The quality of evidence was evaluated using the GRADE score.

## Results

### Study selection

**FIGURE 1:**
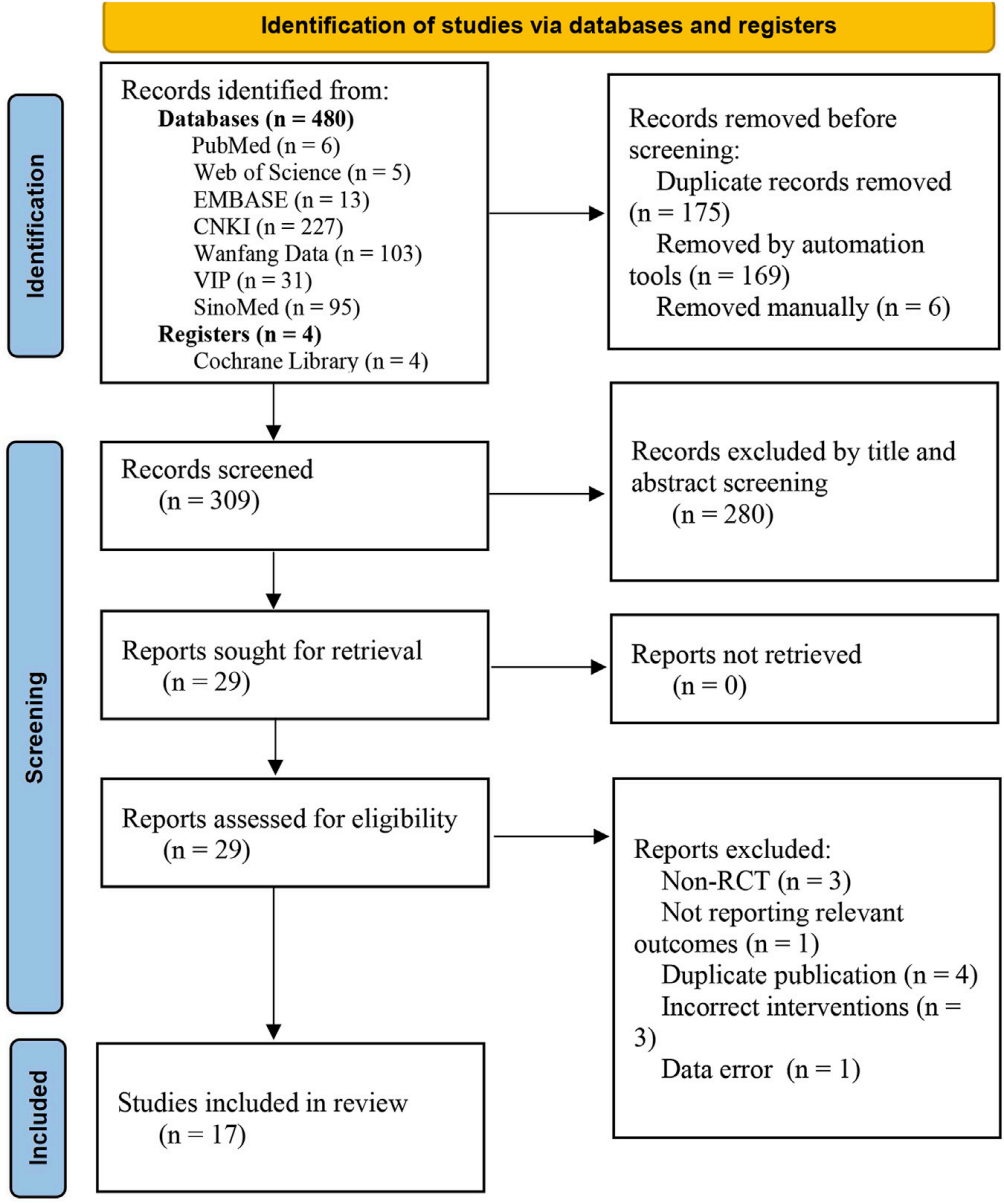
Flowchart of the study selection process.

A total of 484 relevant documents were retrieved using the search strategy. A total of 309 articles were obtained after the software and manual elimination of duplicate studies. Titles and abstracts were read to exclude 280 documents that were not relevant to the theme. The remaining 29 documents were read in full, and 12 non-compliant documents were excluded (three non-RCTs, 1 not reporting relevant outcomes, four duplicate publications, three incorrect interventions, and one data error). Ultimately, 17 studies were included in the meta-analysis. The selection process is illustrated in Figure 1.

### Study characteristics

All 17 included RCTs (Chai et al., 2020; Chen, 2023; Guo, 2018; Jia and Wei, 2022; Jiang et al., 2022; Jin and Ye, 2023; Kan and Yi, 2020; Liang, 2010; Lu and Xiao, 2018; Wang et al., 2017; Wu, 2012; Xie et al., 2015; Yang, 2023; Yang et al., 2007; Yin, 2016; Zheng, 2021; Zou, 2016) were single-center clinical trials. All study sites were located in mainland China. The selected studies were published between 2007 and 2023. A total of 1,366 participants were enrolled in 17 studies, including 683 in the experimental group and 683 in the control group. The maximum and minimum sample sizes were 120 and 56, respectively. The ATDs used in the 16 RCTs (Chai et al., 2020; Chen, 2023; Guo, 2018; Jia and Wei, 2022; Jiang et al., 2022; Jin and Ye, 2023; Kan and Yi, 2020; Liang, 2010; Lu and Xiao, 2018; Wang et al., 2017; Wu, 2012; Xie et al., 2015; Yang, 2023; Yang et al., 2007; Yin, 2016; Zheng, 2021) was methimazole. The ATDs used in one RCT (Zou, 2016) was methimazole in combination with propylthiouracil. The dosage forms of PVL were analyzed: six RCTs (Chai et al., 2020; Chen, 2023; Wu, 2012; Xie et al., 2015; Yang et al., 2007; Zou, 2016) used PVL oral liquid, three RCTs (Guo, 2018; Jia and Wei, 2022; Lu and Xiao, 2018) used PVL capsules, and eight RCTs (Jiang et al., 2022; Jin and Ye, 2023; Kan and Yi, 2020; Liang, 2010; Wang et al., 2017; Yang, 2023; Yin, 2016; Zheng, 2021) used PVL granules. Treatment duration was analyzed: 11 RCTs (Guo, 2018; Jia and Wei, 2022; Jiang et al., 2022; Jin and Ye, 2023; Kan and Yi, 2020; Liang, 2010; Lu and Xiao, 2018; Wang et al., 2017; Yang, 2023; Yin, 2016; Zou, 2016) had a duration of less than 6 months and 6 RCTs (Chai et al., 2020; Chen, 2023; Wu, 2012; Xie et al., 2015; Yang et al., 2007; Zheng, 2021) had a duration of greater than or equal to 6 months. Six RCTs (Chai et al., 2020; Chen, 2023; Guo, 2018; Jin and Ye, 2023; Liang, 2010; Yang, 2023) described described the syndromes to which patients with hyperthyroidism belonged, including two (Chai et al., 2020; Liang, 2010) on the syndrome of exuberant fire of the heart and liver, three (Chen, 2023; Guo, 2018; Yang, 2023) on the syndrome of exuberance of liver fire, and one (Jin and Ye, 2023) on the pattern of qi stagnation and phlegm coagulation. All studies reported comparable baseline characteristics (e.g., patient age, sex, and disease duration). The characteristics of the included studies are summarized in Table 1.

**TABLE 1:** Basic characteristics of the included studies.

Study ID	Sample size (E/C)	Age (year)	Male/Female	Disease duration	Intervention(s)	Comparators	Treatment duration	TCM syndrome	Outcomes
Chai et al. (2020)	50/50	E: 48.27 ± 10.93	E: 18/32	E: 3.2 ± 1.4(y)	XKC oral liquid 10 mL bid + MMI	MMI	48w	Syndrome of effulgent heart-liver fire	1, 2, 3, 4, 5, 10, 11
		C: 45.83 ± 11.65	C: 15/35	C: 3.6 ± 1.8(y)					
Chen (2023)	28/28	E: 37.96 ± 3.46	E: 8/20	E: 2.65 ± 0.63(m)	XKC oral liquid 10 mL bid + MMI	MMI	6m	Intense liver fire pattern	1, 2, 3, 4, 5, 10, 11
		C: 38.23 ± 3.53	C: 7/21	C: 2.63 ± 0.67(m)					
Guo (2018)	30/32	E: 37.73 ± 11.20	E: 9/21	E: 1.56 ± 1.38(m)	XKC capsule 0.7g bid + MMI	MMI	8w	Intense liver fire pattern	1, 2, 3, 11
		C: 35.13 ± 9.65	C: 13/19	C: 2.04 ± 2.52(m)					
Jia and Wei (2022)	32/32	E: 39.06 ± 7.68	E: 11/21	E: 2.32 ± 0.58(y)	XKC capsule 0.7g bid + MMI	MMI	90d	NR	1, 2, 3, 11
		C: 38.46 ± 8.44	C: 10/22	C: 2.23 ± 0.64(y)					
Jiang et al. (2022)	40/40	E: 36.03 ± 7.47	E: 7/33	NR	XKC granule 9g bid + MMI	MMI	2m	NR	1, 2, 3, 11
		C: 35.87 ± 7.24	C: 9/31						
Jin and Ye (2023)	39/39	E: 42.35 ± 7.13	E: 15/24	E: 1.36 ± 0.67(y)	XKC granule 2g bid + MMI	MMI	3m	Pattern of qi stagnating and phlegm congealing	1, 2, 3, 4
		C: 41.20 ± 8.05	C: 13/26	C: 1.41 ± 0.46(y)					
Kan and Yi (2020)	43/43	E: 42.56 ± 5.32	E: 18/25	E: 2.21 ± 1.03(y)	XKC granule 2g bid + MMI	MMI	3m	NR	1, 2, 3, 6, 7, 8, 9, 11
		C: 43.17 ± 5.48	C: 16/27	C: 2.45 ± 0.95(y)					
Liang (2010)	30/30	E: 38.4 ± 12.6	E: 9/21	NR	XKC granule 2g bid + MMI	MMI	12w	Syndrome of effulgent heart-liver fire	1, 2, 3, 11
		C: 39.7 ± 11.8	C: 10/20						
Lu and Xiao (2018)	40/40	E: 36.48 ± 10.19	E: 22/18	E: 0.42 ± 0.22(y)	XKC capsule 0.7g bid + MMI	MMI	90d	NR	1, 2, 3
		C: 37.13 ± 11.56	C: 17/23	C: 0.53 ± 0.14(y)					
Wang et al. (2017)	41/39	38 ± 6	E: 13/28	E: 5.3 ± 1.2(m)	XKC granule 9g bid + MMI	MMI	12w	NR	1, 2, 3, 6, 7, 8, 9
			C: 12/27	C: 5.4 ± 1.4(m)					
Wu (2012)	60/60	E: 32.16 ± 8.59	E: 18/42	E: 70 ± 28(d)	XKC oral liquid 10 mL bid + MMI, 2 months later, adjust it to XKC oral liquid 10 mL qd + MMI	MMI	18m	NR	1, 2, 3, 5, 10
		C: 31.52 ± 8.73	C: 16/44	C: 68 ± 26(d)					
Xie et al. (2015)	43/43	41.3 ± 2.3	NR	NR	XKC oral liquid 5 mL bid + MMI, 2 months later, adjust it to XKC oral liquid 5 mL qd + MMI	MMI	6m	NR	1, 2, 3, 5
Yang (2023)	36/36	E: 37.76 ± 11.44	NR	NR	XKC granule 15g bid + MMI	MMI	8w	Intense liver fire pattern	1, 2, 3, 5, 6, 7,11
		C: 38.66 ± 10.69							
Yang et al (2007)	28/28	40.4 ± 12.3	E: 11/17	NR	XKC oral liquid 10 mL bid + MMI, 2 months later, adjust it to XKC oral liquid 10 mL qd + MMI	MMI	6m	NR	1, 2, 3, 5
			C: 10/18						
Yin (2016)	49/49	E: 37.8 ± 10.2	E: 13/36	E: 1.6 ± 0.5(y)	XKC granule 2g bid + MMI	MMI	90d	NR	1, 2, 3, 11
		C: 37.9 ± 10.4	C: 14/35	C: 1.6 ± 0.6(y)					
Zheng (2021)	49/49	E: 43.69 ± 1.28	E: 22/27	E: 6.80 ± 0.52(m)	XKC granule 9g bid + MMI	MMI	24m	NR	1, 2, 3, 11
		C: 43.70 ± 1.31	C: 20/29	C: 6.77 ± 0.56(m)					
Zou (2016)	45/45	E: 38.5 ± 6.7	E: 11/34	E: 2.4 ± 0.8(m)	XKC oral liquid 10 mL bid + MMI, PTU	MMI, PTU	12w	NR	1, 2, 3, 4, 5
		C: 37.8 ± 7.2	C: 9/36	C: 2.6 ± 0.7(m)					

E, experimental group; C, control group; XKC, xiakucao; MMI, methimazole; PTU, propylthiouracil; NR, not reported; d: day; m, month; y, year; 1, free triiodothyronine (FT3); 2, free thyroxine (FT4); 3, thyroid stimulating hormone (TSH); 4, thyrotropin receptor antibody (TRAb); 5, thyroid gland size; 6, tumor necrosis factor alpha (TNF-α); 7, interleukin-6 (IL-6); 8, interleukin-10 (IL-10); 9, interferon gamma (IFN-γ); 10, relapse rate; 11, adverse events.

### Study quality

**FIGURE 2:**
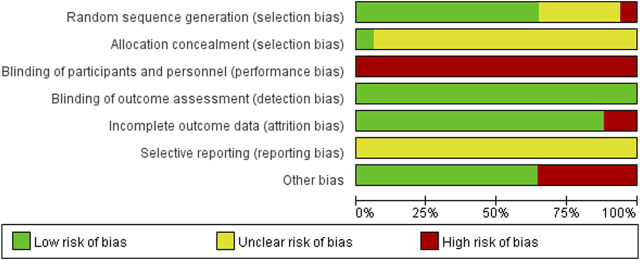
Risk of bias summary.

Eleven RCTs (Chai et al., 2020; Guo, 2018; Jia and Wei, 2022; Jiang et al., 2022; Jin and Ye, 2023; Kan and Yi, 2020; Lu and Xiao, 2018; Xie et al., 2015; Yang et al., 2007; Yang, 2023; Zheng, 2021) reported specific methods of randomization (grouping using the random number table method) and were classified as low-risk. One study (Yin, 2016) was grouped by visit serial number (single or double) and rated as high risk. The remaining five studies (Chen, 2023; Liang, 2010; Wang et al., 2017; Wu, 2012; Zou, 2016) did not describe a specific randomization method and only mentioned randomization, which was rated as unclear. One study (Liang, 2010) used closed envelopes to conceal the distribution scheme and was considered low risk. The allocation concealment methods were not adequately reported in the remaining studies; hence the risk of bias was rated as unclear. None of the studies reported the blinding of personnel and participants who were considered high-risk. Although none of the 17 RCTs mentioned blinding of the outcome assessment, the outcomes included were all objective indicators and were therefore considered low-risk. In terms of incomplete outcome data, two studies (Guo, 2018; Yang, 2023) reported that participants were lost to follow-up, which may have affected the authenticity of the results. Therefore, we rated them as high-risk. For selective reporting, none of the protocols included in the study were retrieved from the Clinical Trials Registry platform and rated as unclear. Regarding other biases, six studies (Guo, 2018; Liang, 2010; Lu and Xiao, 2018; Wang et al., 2017; Wu, 2012; Yin, 2016) explicitly included patients with GD but did not report the key outcome of TRAb. These studies were rated as high risk. The risk of bias assessment is shown in Figure 2.

## Thyroid function and antibody

### Free triiodothyronine (FT3)

**FIGURE 3:**
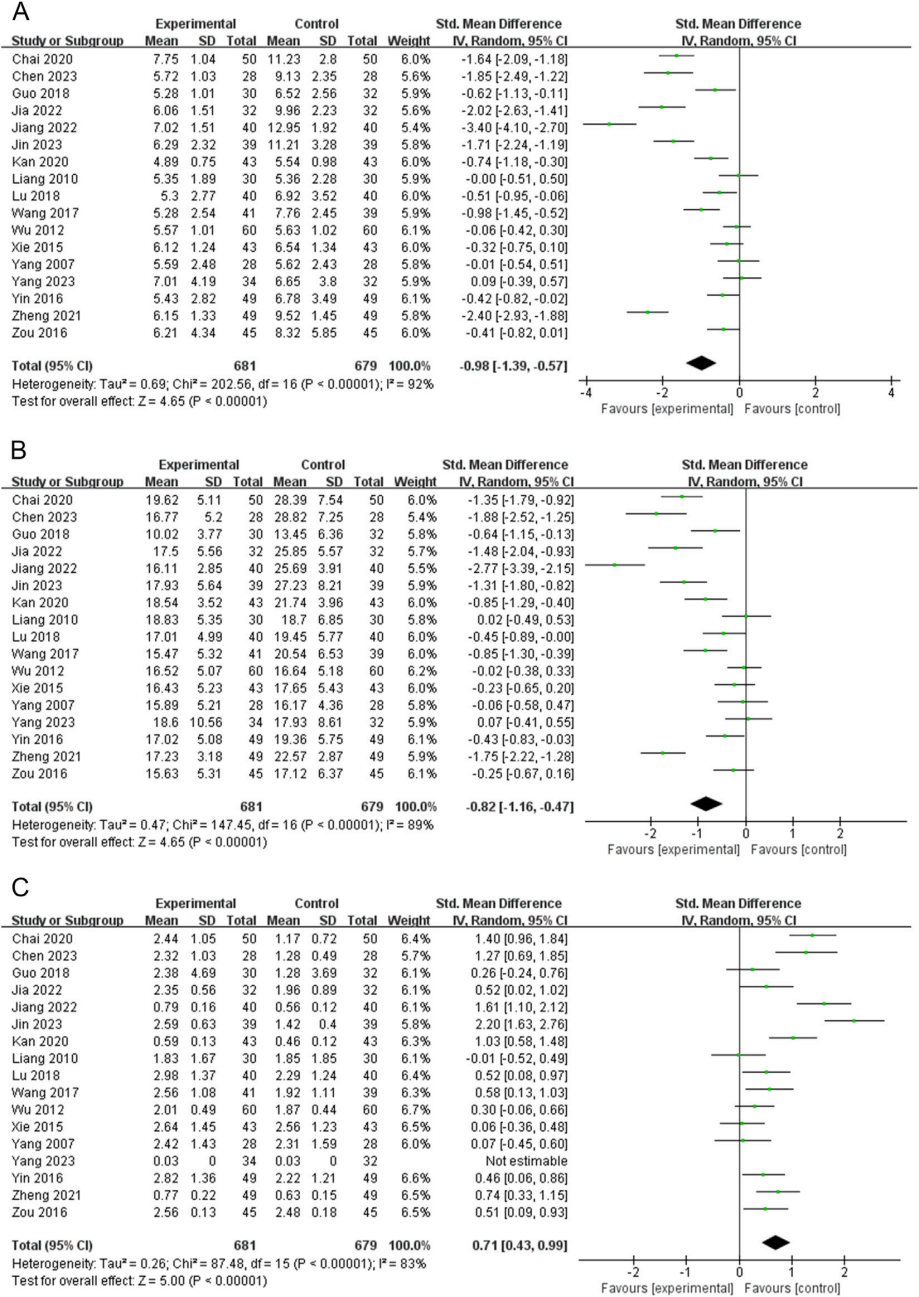
Forest plots for the meta-analysis of **(A)** free triiodothyronine (FT3), **(B)** free thyroxine (FT4), and **(C)** thyroid-stimulating hormone (TSH).

Seventeen RCTs (1,360 subjects) reported FT3 levels (Chai et al., 2020; Chen, 2023; Guo, 2018; Jia and Wei, 2022; Jiang et al., 2022; Jin and Ye, 2023; Kan and Yi, 2020; Liang, 2010; Lu and Xiao, 2018; Wang et al., 2017; Wu, 2012; Xie et al., 2015; Yang, 2023; Yang et al., 2007; Yin, 2016; Zheng, 2021; Zou, 2016). The PVL dosage forms in 7 RCTs were oral liquids, 3 RCTs were capsules, and 7 RCTs were granules. The heterogeneity test indicated high heterogeneity among the studies (*P* < 0.00001; I^2^ = 92%). Therefore, meta-analysis was performed using a random-effects model. The combination of PVL preparations was more effective in reducing FT3 levels than treatment with ATDs alone [SMD = −0.98, 95%CI (−1.39, −0.57), *P* < 0.00001] (Figure 3A). To explore the factors influencing heterogeneity, we performed meta-regression and subgroup analyses of the included studies according to the duration of intervention, type of PVL preparation, and sample size. Neither meta-regression nor subgroup analysis explained the source of heterogeneity (Supplementary Figure S1; Supplementary Tables S4, S6).

### Free thyroxine (FT4)

FT4 levels were reported in 17 RCTs (1,360 subjects) (Chai et al., 2020; Chen, 2023; Guo, 2018; Jia and Wei, 2022; Jiang et al., 2022; Jin and Ye, 2023; Kan and Yi, 2020; Liang, 2010; Lu and Xiao, 2018; Wang et al., 2017; Wu, 2012; Xie et al., 2015; Yang, 2023; Yang et al., 2007; Yin, 2016; Zheng, 2021; Zou, 2016). The PVL dosage forms in 7 RCTs were oral liquids, 3 RCTs were capsules, and 7 RCTs were granules. The heterogeneity test indicated high heterogeneity among the studies (*P* < 0.00001, I^2^ = 89%); therefore, meta-analysis was performed using a random-effects model. Treatment with PVL preparations in combination with ATDs was superior to treatment with ATDs alone in reducing FT4 levels [SMD = −0.82, 95% CI (−1.16, −0.47), *P* < 0.00001] (Figure 3B). Heterogeneity between the studies was not explained by meta-regression or subgroup analyses (Supplementary Figure S2; Supplementary Tables S4, S6).

### Thyroid-stimulating hormone (TSH)

Seventeen RCTs (1,360 subjects) reported the TSH levels (Chai et al., 2020; Chen, 2023; Guo, 2018; Jia and Wei, 2022; Jiang et al., 2022; Jin and Ye, 2023; Kan and Yi, 2020; Liang, 2010; Lu and Xiao, 2018; Wang et al., 2017; Wu, 2012; Xie et al., 2015; Yang, 2023; Yang et al., 2007; Yin, 2016; Zheng, 2021; Zou, 2016). The heterogeneity test indicated high heterogeneity among the studies (*P* < 0.00001, I^2^ = 83%); therefore, a meta-analysis was performed using a random-effects model. The combination of PVL preparations was more effective in improving TSH levels than treatment with ATDs alone [SMD = 0.71, 95%CI (0.43, 0.99), *P* < 0.00001] (Figure 3C). The subgroup analysis showed significantly less heterogeneity in the PVL capsule subgroup (I^2^ = 0%). This suggests that the type of PVL preparation may be an influencing factor of heterogeneity (Supplementary Figure S3; Supplementary Table S4). The relatively consistent intervention regimen in the PVL capsule subgroup (such as the dosage, manufacturer, and specifications of the PVL capsules) throughout the study may contribute to reducing heterogeneity.

### Thyrotropin receptor antibody (TRAb)

**FIGURE 4:**
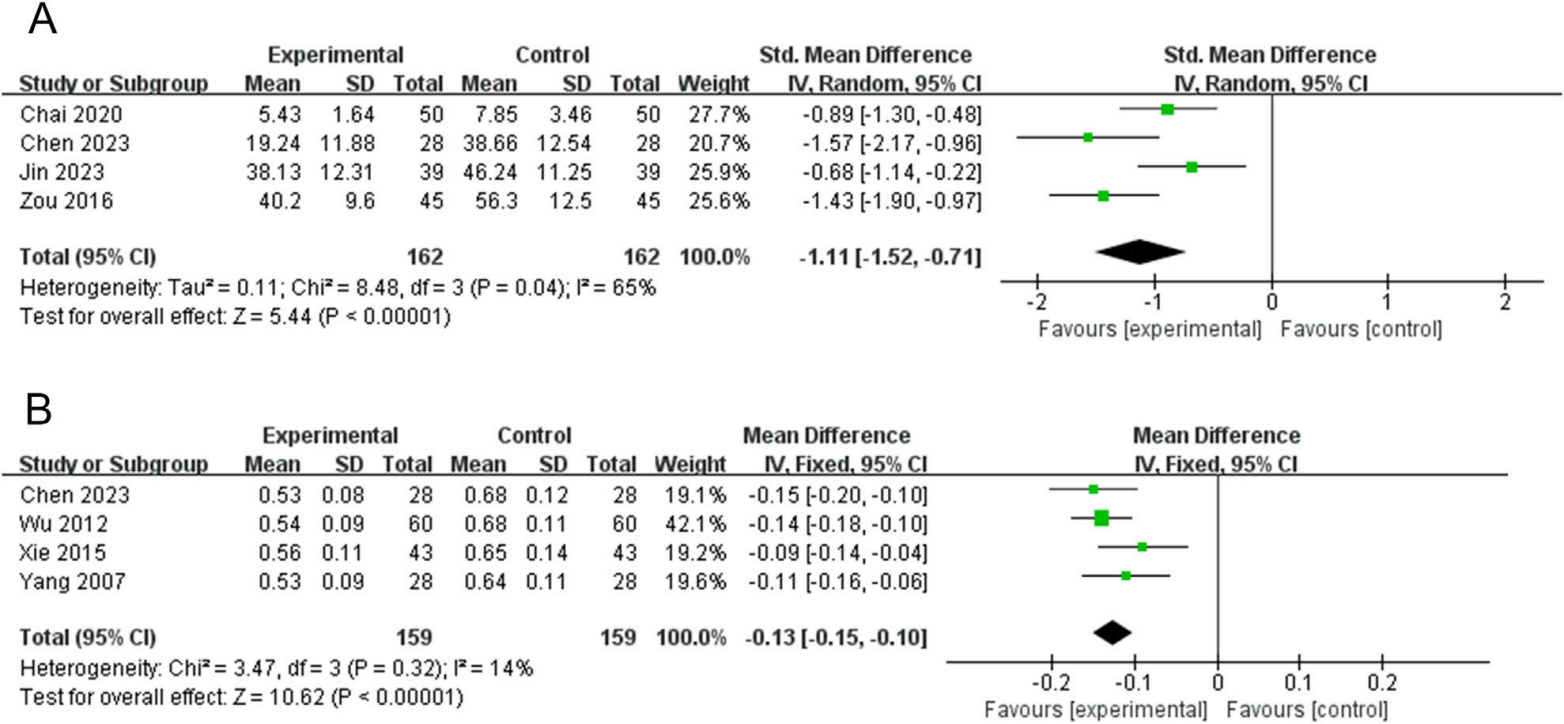
Forest plots for the meta-analysis of **(A)** thyrotropin receptor antibody (TRAb) and **(B)** thyroid isthmus thickness (TIT).

TRAb was reported in four RCTs (324 participants) (Chai et al., 2020; Chen, 2023; Jin and Ye, 2023; Zou, 2016). The PVL dosage forms in the three RCTs were oral liquids, and 1 RCT was granules. Heterogeneity between the studies was significant (*P* = 0.04, I^2^ = 65%); therefore, a meta-analysis was performed using a random-effects model. Treatment with PVL preparations in combination with ATDs was superior to treatment with ATDs alone in reducing TRAb levels [SMD = −1.11, 95%CI (−1.52, −0.71), *P* < 0.00001] (Figure 4A). Heterogeneity among the studies was not explained by meta-regression or subgroup analyses (Supplementary Figure S4; Supplementary Table S4, S6).

## Thyroid gland size

### Thyroid isthmus thickness (TIT)

TIT was reported in 4 RCTs (318 participants) (Chen, 2023; Wu, 2012; Xie et al., 2015; Yang et al., 2007). The PVL dosage forms used in all four RCTs were oral liquids. There was no significant heterogeneity among the studies (*P* = 0.32, I^2^ = 14%). Therefore, meta-analysis was performed using a fixed-effects model. The combination of PVL and oral liquid was effective in reducing TIT compared to treatment with ATDs alone [MD = −0.13, 95%CI (−0.15, −0.10), *P* < 0.00001] (Figure 4B).

### Width of left thyroid lobe (WLTL)

**FIGURE 5:**
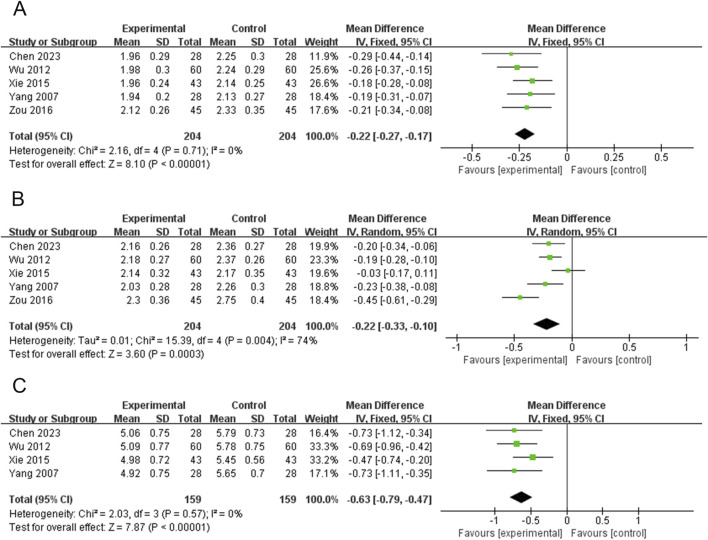
Forest plots for the meta-analysis of **(A)** width of left thyroid lobe (WLTL), **(B)** thickness of left thyroid lobe (TLTL), and **(C)** length of left thyroid lobe (LLTL).

Five RCTs (408 participants) reported on WLTL (Chen, 2023; Wu, 2012; Xie et al., 2015; Yang et al., 2007; Zou, 2016). The PVL dosage forms used in all five RCTs were oral liquids. No heterogeneity was observed among the studies (*P* = 0.71, I^2^ = 0). Therefore, meta-analysis was performed using a fixed-effects model. Treatment with PVL oral liquid in combination with ATDs was superior to treatment with ATDs alone in reducing WLTL [MD = −0.22, 95%CI (−0.27, −0.17), *P* < 0.00001] (Figure 5A).

### Thickness of left thyroid lobe (TLTL)

Five RCTs (408 participants) reported on TLTL (Chen, 2023; Wu, 2012; Xie et al., 2015; Yang et al., 2007; Zou, 2016). The PVL dosage forms used in all five RCTs were oral liquids. There was significant heterogeneity among the studies (*P* = 0.004, I^2^ = 74%); therefore, meta-analysis was performed using a random-effects model. Treatment with PVL plus ATDs was superior to treatment with ATDs alone in attenuating TLTL [MD = −0.22, 95% CI (−0.33, −0.10), *P* = 0.0003] (Figure 5B). Subgroup analysis showed significantly less heterogeneity in the subgroup with sample sizes of <80 (I^2^ = 0%). This suggests that the sample size may be one of the factors influencing heterogeneity (Supplementary Figur S5; Supplementary Table S4).

### Length of left thyroid lobe (LLTL)

Four RCTs (318 participants) reported on LLTL (Chen, 2023; Wu, 2012; Xie et al., 2015; Yang et al., 2007). Meta-analysis was performed using a fixed-effects model because there was no heterogeneity among the studies (*P* = 0.57, I^2^ = 0%). Treatment with PVL preparations in combination with ATDs was superior to treatment with ATDs alone in attenuating LLTL [MD = −0.63, 95%CI (−0.79, −0.47), *P* < 0.00001] (Figure 5C).

### Width of right thyroid lobe (WRTL)

**FIGURE 6:**
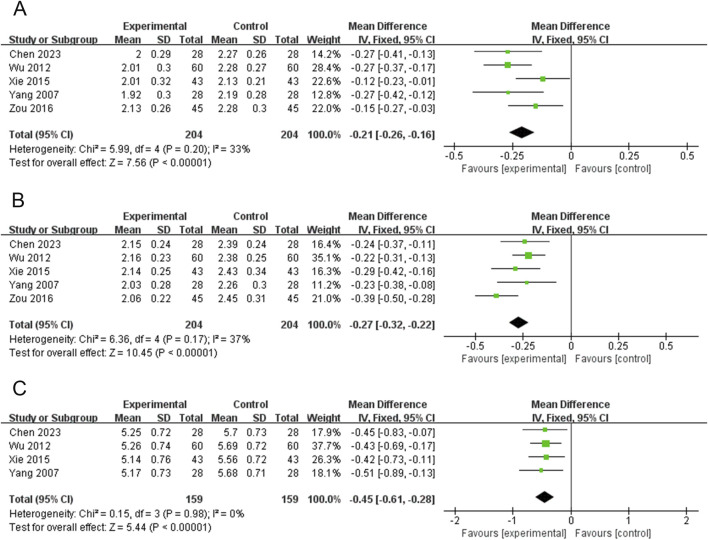
Forest plots for the meta-analysis of **(A)** width of right thyroid lobe (WRTL), **(B)** thickness of right thyroid lobe (TRTL), and **(C)** length of right thyroid lobe (LRTL).

Five RCTs (408 participants) reported on WRTL (Chen, 2023; Wu, 2012; Xie et al., 2015; Yang et al., 2007; Zou, 2016). The PVL dosage forms used in all five RCTs were oral liquids. There was no significant heterogeneity among the studies (*P* = 0.20, I^2^ = 33%); therefore, the effect sizes were combined using a fixed-effects model. Treatment with PVL plus ATDs was superior to treatment with ATDs alone in attenuating WRTL [MD = −0.21, 95%CI (−0.26, −0.16), *P* < 0.00001] (Figure 6A).

### Thickness of right thyroid lobe (TRTL)

Five RCTs (408 participants) reported on TRTL (Chen, 2023; Wu, 2012; Xie et al., 2015; Yang et al., 2007; Zou, 2016). The PVL dosage forms used in all five RCTs were oral liquids. There was no significant heterogeneity among the studies (*P* = 0.17, I^2^ = 37%). Therefore, meta-analysis was performed using a fixed-effects model. Treatment with PVL in combination with ATDs was more effective in reducing TRTL than ATDs alone [MD = −0.27, 95%CI (−0.32, −0.22), *P* < 0.00001] (Figure 6B).

### Length of right thyroid lobe (LRTL)

Four RCTs (318 participants) reported on LRTL (Chen, 2023; Wu, 2012; Xie et al., 2015; Yang et al., 2007). The results of the heterogeneity test showed good homogeneity among the studies (*P* = 0.98, I^2^ = 0%). Therefore, a meta-analysis was performed using a fixed-effects model. PVL oral liquid combined with ATDs treatment was superior to ATDs treatment alone in reducing LRTL [MD = −0.45, 95%CI (−0.61, −0.28), *P* < 0.00001] (Figure 6C).

## Cytokine

### Tumor necrosis factor-α (TNF-α)

**FIGURE 7:**
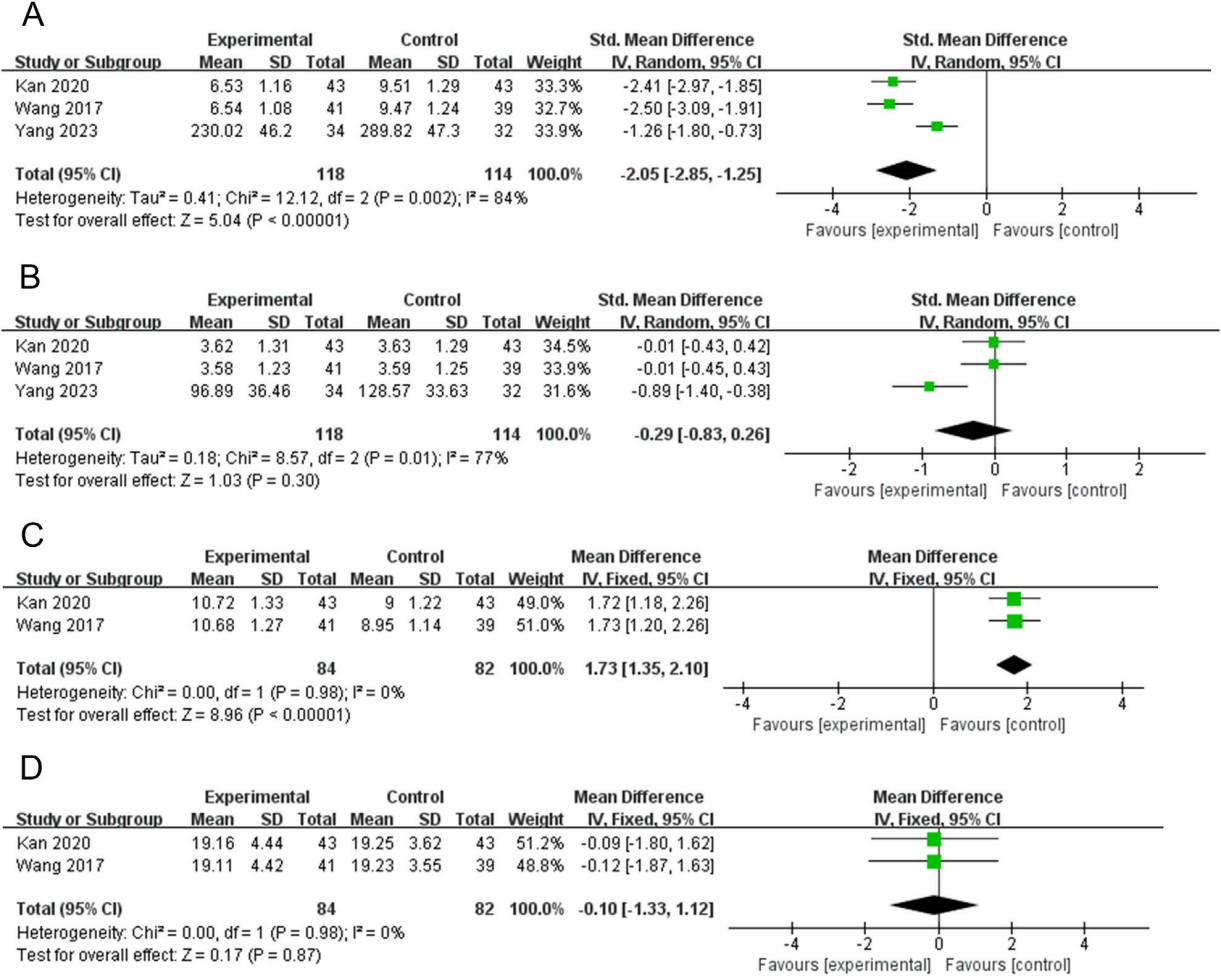
Forest plots for the meta-analysis of **(A)** tumor necrosis factor-α (TNF-α), **(B)** interleukin-6 (IL-6), **(C)** interleukin-10 (IL-10), and **(D)** interferon gamma (IFN-γ).

Three RCTs (232 participants) reported on TNF-α (Kan and Yi, 2020; Wang et al., 2017; Yang, 2023). The PVL dosage forms used in all three RCTs were granules. The heterogeneity among the studies was significant (*P* = 0.002, I^2^ = 84%). Therefore, meta-analysis was performed using a random-effects model. The combination of PVL granules significantly reduced TNF-α compared with treatment with ATDs alone [SMD = −2.05, 95%CI (−2.85, −1.25), *P* < 0.00001] (Figure 7A). Subgroup analysis showed significantly less heterogeneity in the subgroup with a sample size of no less than 80 (I^2^ = 0%). Thus, the sample size may be a potential source of heterogeneity (Supplementary Figure S6A; Supplementary Table S4).

### Interleukin-6 (IL-6)

Three RCTs (232 participants) reported IL-6 levels (Kan and Yi, 2020; Wang et al., 2017; Yang, 2023). The PVL dosage forms used in all three RCTs were granules. The heterogeneity test revealed a high degree of heterogeneity among the studies (*P* = 0.01, I^2^ = 77%); therefore, the random-effects model was used to combine effect sizes. Meta-analysis showed that treatment with PVL granules plus ATDs had no significant effect on IL-6 levels compared to treatment with ATDs alone [SMD = −0.29, 95% CI (−0.83, 0.26), *P* = 0.30] (Figure 7B). In the subgroup with a sample size of no less than 80, I^2^ decreased from 77% to 0%. This suggests that sample size may be a source of heterogeneity (Supplementary Figure S6B; Supplementary Table S4).

### Interleukin-10 (IL-10)

Two RCTs (166 participants) reported IL-10 levels (Kan and Yi, 2020; Wang et al., 2017). The PVL dosage forms used in all 2 RCTs were granules. The heterogeneity test revealed no heterogeneity between the studies (*P* = 0.98, I^2^ = 0%); therefore, a meta-analysis was performed using a fixed-effects model. Treatment with PVL granules combined with ATDs was superior to treatment with ATDs alone in elevating IL-10 levels [MD = 1.73, 95%CI (1.35, 2.10), *P* < 0.00001] (Figure 7C).

### Interferon gamma (IFN-γ)

Two RCTs (166 participants) reported IFN-γ levels (Kan and Yi, 2020; Wang et al., 2017). The PVL dosage forms used in all 2 RCTs were granules. There was no heterogeneity among the studies (*P* = 0.98, I^2^ = 0%); therefore, the effect sizes were combined using a fixed-effects model. Meta-analysis showed that treatment with PVL granules plus ATDs had no significant effect on IFN-γ levels compared with treatment with ATDs alone [MD = −0.10, 95% CI (−1.33, 1.12), P = 0.87] (Figure 7D).

### Relapse rate

**FIGURE 8:**
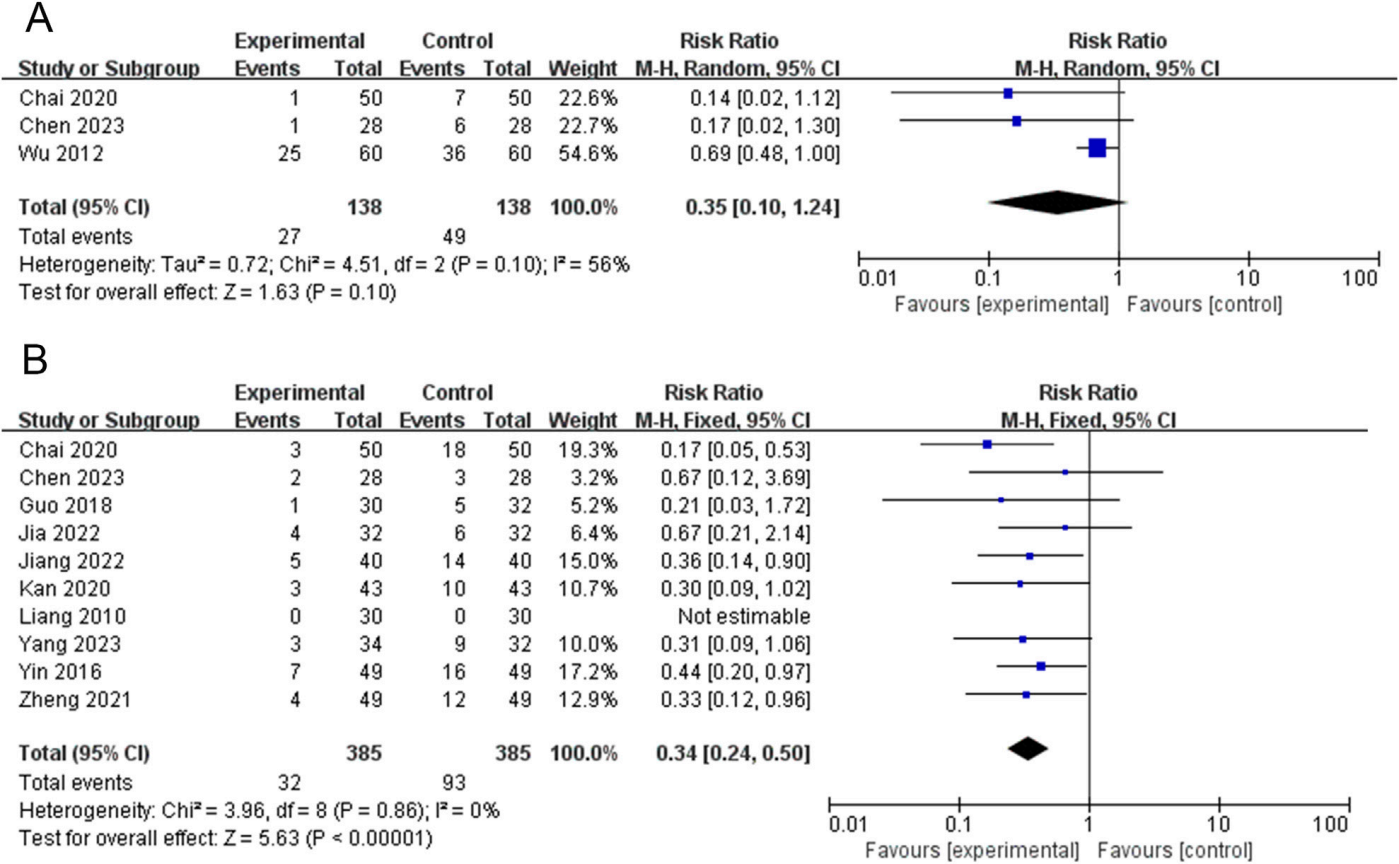
Forest plots for the meta-analysis of **(A)** relapse rate and **(B)** adverse events.

Three RCTs (276 participants) reported recurrence rates (Chai et al., 2020; Chen, 2023; Wu, 2012). The PVL dosage forms used in all three RCTs were oral liquids. Owing to the heterogeneity among the studies (*P* = 0.10, I^2^ = 56%), a meta-analysis was performed using a random-effects model. The results showed no significant difference in the recurrence rate between the experimental group and control group [RR = 0.35, 95%CI (0.10, 1.24), *P* = 0.10] (Figure 8A).

### Adverse events

Ten RCTs (770 participants) reported adverse events (Chai et al., 2020; Chen, 2023; Guo, 2018; Jia and Wei, 2022; Jiang et al., 2022; Kan and Yi, 2020; Liang, 2010; Yang, 2023; Yin, 2016; Zheng, 2021). The results of the heterogeneity test showed good homogeneity among the studies (*P* = 0.86, I^2^ = 0%); therefore, a meta-analysis was performed using a fixed-effects model. Treatment with PVL preparations in combination with ATDs was superior to treatment with ATDs alone in reducing adverse events [RR = 0.34, 95%CI (0.24, 0.50), *P* < 0.00001] (Figure 8B). One RCT (Liang, 2010) reported no adverse events. Nine RCTs (Chai et al., 2020; Chen, 2023; Guo, 2018; Jia and Wei, 2022; Jiang et al., 2022; Kan and Yi, 2020; Yang, 2023; Yin, 2016; Zheng, 2021) reported reported adverse reactions in 32 patients in the experimental group, including leukopenia in 3 cases, liver function abnormalities in 8 cases, secondary hypothyroidism in 1 case, gastrointestinal reactions such as nausea, vomiting, and diarrhea in 7 cases, burning sensation in the mouth in 1 case, skin rash in 6 cases, palpitations in 1 case, dizziness and headache in 2 cases, and anxiety and depression in 3 cases. Adverse reactions occurred in 93 patients in the control group, including 14 cases of leukopenia; 24 cases of liver function abnormalities; 5 cases of secondary hypothyroidism; 12 cases of gastrointestinal reactions such as nausea, vomiting, and diarrhea; 1 case of burning sensation in the mouth; 21 cases of skin rash; 1 case of palpitations; 9 cases of dizziness and headache; and 6 cases of anxiety and depression. The adverse reactions are detailed in Supplementary Table S5.

### Meta-regression

In this study, we performed univariate and multivariate meta-regression analyses of the FT3, FT4, and TSH levels. None of the three factors (duration of intervention, type of PVL preparation, and sample size) were statistically significant. Supplementary Table S6 presents the results of meta-regression.

### Sensitivity analysis

In the sensitivity analysis of the recurrence rate, the meta-analysis results changed from [RR = 0.35, 95%CI (0.10, 1.24), *P* = 0.10] to [RR = 0.15, 95%CI (0.04, 0.66), *P* = 0.01] after excluding the study (Wu, 2012). The results of the sensitivity analysis for the remaining outcomes (e.g., FT3, FT4, TSH, and TRAb) were relatively robust. Supplementary Table S7 presents the sensitivity analysis results.

### Publication bias

**FIGURE 9:**
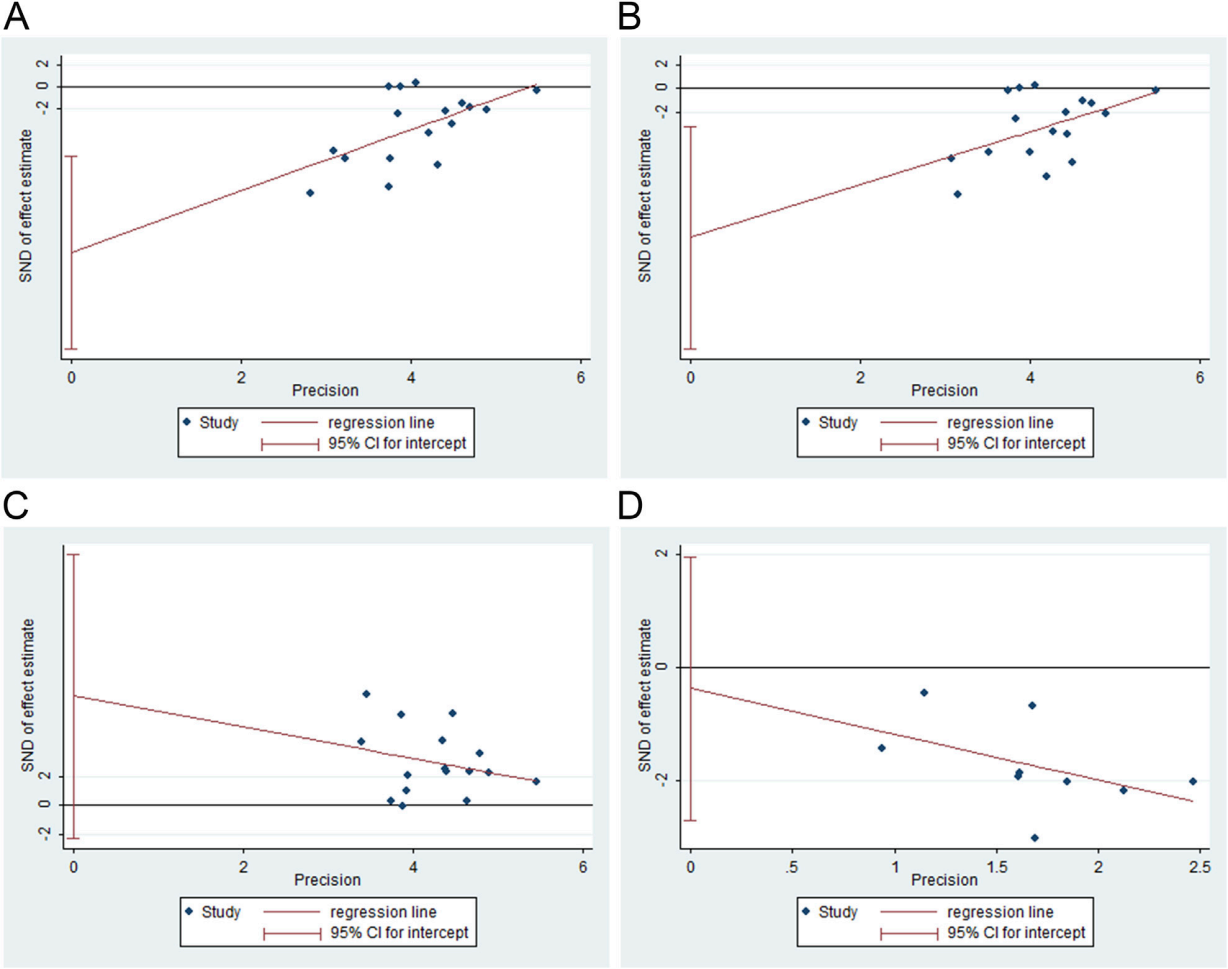
Publication bias analysis. **(A)** free triiodothyronine (FT3), **(B)** free thyroxine (FT4), **(C)** thyroid-stimulating hormone (TSH), and **(D)** adverse events.

FT3, FT4, and TSH levels and adverse events were assessed for publication bias using Egger’s test with Stata software. The results showed publication bias for FT3 (t = −3.66, *P* = 0.002), FT4 (t = −2.86, *P* = 0.012), and TSH (t = 1.65, *P* = 0.122). No publication bias was observed for adverse events (t = −0.38, *P* = 0.713). Figure 9 shows the results for publication bias.

### GRADE analysis

GRADE analysis was performed for each of the included outcomes. Adverse events were considered moderate-quality evidence. TSH was considered low-quality evidence. The remaining outcomes, such as FT3, FT4, TRAb, WLTL, TLTL, LLTL, TIT, WRTL, TRTL, LRTL, TNF-α, IL-6, IL-10, IFN-γ, and relapse rate, were considered very low-quality evidence. Supplementary Table S8 shows the results of the GRADE analysis.

## Discussion

### Summary of results

Thyrotoxicosis is a clinical syndrome characterized by excess circulating thyroid hormones due to various causes, resulting in increased excitability and metabolic hyperactivity in the nervous, circulatory, and digestive systems. Hyperthyroidism is a well-known etiology of thyrotoxicosis. Among these, Graves hyperthyroidism is the most common. GD can damage several organs of the body. Graves’ ophthalmopathy (GO) of varying severity has been reported in 25%–40% of patients with Graves’ hyperthyroidism (Bartalena et al., 2020). GD can cause fetal, neonatal, and maternal complications (Illouz et al., 2018). Furthermore, there have been case reports of severe pulmonary hypertension, cholestatic liver injury, heart failure, and pancytopenia in individual patients (Nigussie et al., 2020; Sabra et al., 2024). The use of Chinese herbal medicines for the treatment of hyperthyroidism has attracted increasing attention in recent years. Botanical drugs can improve the efficacy of patients with GD, improve thyroid function, and reduce TRAb and thyroid peroxidase antibody levels (Yang et al., 2024). PVL is a popular botanical drug, that is found in Europe, Asia, Africa, North America, and Australia (Wang et al., 2019). Previous studies have shown the potential therapeutic value of PVL and its extracts in GD (Zhang et al., 2024), subacute thyroiditis (Li et al., 2019), Hashimoto’s thyroiditis (Chen et al., 2020), and thyroid cancer (Yu et al., 2021; Song et al., 2021). PVL has natural immunomodulatory activity (Chen et al., 2024). It may exert a protective effect on thyroid cells by inhibiting innate and adaptive immune responses and inducing cell death (Chen et al., 2020). In rats with experimental autoimmune thyroiditis (EAT), PVL increased 5-hydroxytryptamine levels and inhibited Th17 cell differentiation (Zhang et al., 2022). HMGB1 and Toll-like receptors are important biomarkers and potential therapeutic targets for autoimmune diseases (Ren et al., 2023). Inhibition of the HMGB1/TLR9/MyD88 pathway is also an important mechanism by which PV attenuates EAT (Guo et al., 2021). In addition, PV can alleviate autoimmune thyroiditis and improve pregnancy outcomes by suppressing Th1/Th17 immune responses and inducing Treg cell proliferation (Zhu et al., 2022).

The FT3, FT4, and TSH levels are the main indicators for the clinical diagnosis of hyperthyroidism. TRAb contributes to the etiology of thyrotoxicosis. It is an important indicator for the diagnosis of GD as well as for determining the prognosis of GD and discontinuation of ATD therapy. A study with seventeen trials on PVL preparations in combination with ATDs for the treatment of hyperthyroidism was included in this review. We quantified thyroid function (FT3, FT4, TSH), thyrotropin receptor antibody (TRAb), thyroid gland size (WLTL, TLTL, LLTL, TIT, WRTL, TRTL, LRTL), cytokines (TNF-α, IL-6, IL-10, IFN-γ), relapse rate, and adverse events to comprehensively evaluate its efficacy and safety. We found that the combination of PVL preparations reduced FT3, FT4, and TRAb levels, increased TSH levels, and reduced goiter in patients with hyperthyroidism compared to treatment with ATDs alone. PVL effectively reduced FT3, FT4, and TRAb levels, goiter volume, and TSH levels in hyperthyroid patients (Zhu et al., 2021). A recent network meta-analysis showed that PVL granules were the most effective in lowering FT3 and FT4 levels in patients with GD compared with other botanical drugs, whereas PVL oral liquid performed the best in lowering TRAb and TPOAb levels in patients with GD (Yang et al., 2024). Wu et al. (2024) also found that PVL preparations combined with biomedicine therapy (ATDs or I^131^ treatment) were more effective than biomedicine therapy alone at improving thyroid hormone levels, thyroid antibody titers, and thyroid size in patients with GD. The results of this study show that PVL preparations could reduce the side effects of ATDs treatment. Current studies indicate that PVL has a high clinical safety profile for use. Acute toxicity studies showed that during the 14-day observation period, there were no deaths and no apparent signs of toxicity in the animals. Acute toxicity median lethal dose (LD50) of PVL extract administered by gavage to mice >21.5 g/kg. Subchronic toxicity tests did not reveal any significant toxic effects or target organ damage in PVL extract. Maximum unobserved adverse reaction dose >11.73 g/kg (equivalent to a PVL botanical drug dose of 92.58 g/kg) (Zhao et al., 2017). The results of acute toxicity experiments by Zhong et al. (2001) showed that the oral LD50 of PVL granules in mice was 95.794 g/kg, which was 119.74 times the clinical dose. Rats were continuously injected intramuscularly with PVL injection (0.2 mL/100 g) for 4 weeks, and no significant toxicity was observed at the end of administration or within 2 weeks of withdrawal (Hou et al., 2015). According to the record of adverse reactions in the last 5 years after the launch of the PVL oral solution, 105 cases of adverse reactions occurred in more than one million patients who used the drug. Specifically, 90 patients experienced gastrointestinal adverse reactions (nausea, abdominal pain, diarrhea, etc.), 32 patients experienced skin itching and rash, and 14 patients experienced neurological adverse reactions (mainly dizziness). The above adverse reactions resolved after discontinuation of the drug (Experts of Clinical Application of Xiakucao Oral Liquid, 2020). Moreover, the addition of PVL preparations to ATDs therapy was also more effective in lowering TNF-α levels and elevating IL-10 levels in patients with hyperthyroidism than ATDs alone. Animal studies confirmed that PVL promotes the expansion of splenic Tregs and increases IL-10 production (Qiu et al., 2020). Guo et al. (2021) observed that PVL reduced the production of inflammatory cytokines TNF-α, IL-6, IL-1β, and others both *in vivo* and *ex vivo*. However, in evaluating the effects of PVL preparations on relapse rate, IL-6, and IFN-γ, the results of this study have not been able to demonstrate a significant reduction in relapse rate, IL-6, and IFN-γ levels in patients with hyperthyroidism.

Approximately 200 metabolites have been isolated from PVL, mainly triterpenoids, flavonoids, steroids, coumarins, phenylpropanoids, polysaccharides, and volatile oils (Wang et al., 2019). Network pharmacological studies have shown that PVL contains luteolin, quercetin, β-sitosterol, kaempferol, stigmasterol, mulberry pigment, and other metabolites. The key targets of PVL for the treatment of GD are AKT1, IL-6, TNF, VEGFA, TP53, IL-10, CXCL8, CCL2, MMP-9, and IL-1β. The TNF, HIF-1, PI3K/AKT, and TLR signaling pathways are potential pathways for the PVL treatment of GD (Li and Wei, 2021). Polysaccharides, which are metabolites isolated from PVL, can be used as potential antioxidants and immunomodulators (Li et al., 2015). PLV polysaccharides improve symptoms and thyroid function in mice with GD. Mechanistically, PLV polysaccharides regulate the Ras/Raf/MAPK/ERK signaling pathway by inhibiting the phosphorylation of Raf, MEK1/2, and ERK1/2, which inhibits the downstream expression of IFN-γ, IL-6, IL-17, and TGF-β1 (Xiang et al., 2020). Luteolin is an important chemical component of the PVL used for GD treatment. Zhang et al. (2024) found that PVL and luteolin can treat GD by restoring the balance between Tfh/Tfr cells and alleviating oxidative stress. This effect was associated with the activation of the Nrf2/HO-1 pathway and inhibition of the PI3K/AKT pathway. The PI3K/AKT signaling pathway is also been shown to be involved in the pathogenesis of GO. In thyroid-eye disease, TSHR signaling directly stimulates the proliferation of orbital fibroblasts through the PI3K/AKT pathway (Woeller et al., 2019). Selective PI3Kδ inhibitors suppress proinflammatory cytokine production and adipogenesis in GO orbital fibroblasts *in vitro* (Ko et al., 2018). Notably, Zhang et al. (2020) reported that PVL treatment of thyroid-associated ophthalmopathy (TAO) may be achieved by inhibiting inflammation and proliferation, as well as by promoting apoptosis through the PI3K/AKT pathway. Another *in vitro* study confirmed that PVL polysaccharides exerted anti-TAO effects by inhibiting orbital fibroblast proliferation and promoting apoptosis (Li et al., 2020a).

### Strength and limitation

To our knowledge, this is the first systematic review and meta-analysis of PVL preparations for the treatment of hyperthyroidism. Two published meta-analysis of PVL for hyperthyroidism or GD (Wu et al., 2024; Zhu et al., 2021). The publication languages used were Chinese. Regarding the study population, this study included patients with causes of hyperthyroidism other than GD, in contrast to the study by Wu et al. (2024). Unlike the study by Zhu et al. (2021), the present study included other PVL preparations, such as PVL capsules and granules, in addition to the PVL oral liquid. In terms of control measures, unlike the studies by Wu et al. (2024) and Zhu et al. (2021), the intervention in the control group in this study was limited to ATDs treatment and did not include I^131^ treatment or ATDs combined with Se yeast treatment. In terms of outcome, TNF-α, IL-6, IL-10, IFN-γ, and relapse rate were added in this study to comprehensively evaluate the efficacy of the PVL preparations in intervening in hyperthyroidism. Regarding the number of included studies, this review included more RCTs (Wu et al. (2024) included 8 RCTs; Zhu et al. (2021) included 8 RCTs). For statistical analysis, the study was powered by subgroup analysis, meta-regression, Egger’s test, and GRADE quality of evidence.

However, there are some limitations. (1) The number of included studies for some of the outcomes (e.g., TRAb, TNF-α, IL-6, IL-10, IFN-γ, etc.) was small. These outcomes lack persuasiveness due to small sample sizes and/or variability in study methodologies. (2) The included studies were all single-center clinical trials conducted in mainland China, and extrapolation of the results was limited. (3) The language of publication of the included studies was limited to English and Chinese, which may have resulted in incomplete searches. (4) The methodological quality of the included studies is poor. Some studies did not describe the randomization grouping method in detail. None of the studies mentioned allocation concealment, blinding or clinical trial registration. (5) The short treatment duration in some studies made it difficult to assess the long-term efficacy of PVL preparations. (6) Current studies are not sufficiently comprehensive to consider the safety of commercial Chinese polyherbal preparation. 41% of the trials did not report any adverse events. (7) Some studies did not report the disease duration or sex ratios. Therefore, we could not perform a subgroup analysis based on disease duration and sex to further explore the sources of heterogeneity in certain outcomes with high heterogeneity.

### Implication for future studies

The results of this study provide evidence for the clinical use of PVL preparations for the treatment of hyperthyroidism. Given the problems with the current study, it is recommended that future researchers: (1) Improve the clinical trial design. Examples include the method of generating randomized sequences, allocation concealment, blinding, withdrawal, and loss to follow-up. (2) Strict adherence to the Consolidated Standards of Reporting Trials (CONSORT) statement for standardized and normalized clinical trial reporting. (3) TRAb are characteristic antibodies against GD. This is important for diagnosis, assessment of disease activity, and evaluation of the timing of drug discontinuation. Additionally, it is the most important indicator for predicting GD recurrence. The inclusion of TRAb as an outcome measure is recommended in future clinical trials on GD hyperthyroidism. (4) Attention should be paid to the safety of Chinese herbal medicines in clinical trials. The reporting of adverse reactions must be detailed and complete, including evaluation indicators, specific measurement methods, and acquisition of relevant data. Rather than simply reporting the presence or absence of adverse effects. The reliability of the results of systematic reviews has been improved by conducting high-quality clinical trials.

## Conclusion

In conclusion, PVL preparations combined with ATDs for the treatment of hyperthyroidism improve thyroid function, reduce TRAb levels and goiter size, and inhibit inflammatory responses. In terms of safety, PVL preparations not only do not increase the incidence of adverse events but also mitigate the adverse reactions caused by the use of ATDs. However, owing to the small sample size of the included studies, poor methodological quality, and publication bias. Therefore, the above conclusions need to be further validated by larger, multicenter, high-quality RCTs with long-term follow-up.

## Data Availability

The original contributions presented in the study are included in the article/Supplementary Material, further inquiries can be directed to the corresponding authors.
