# Supplementary material for: Efficacy and safety of Prunella vulgaris L. combined with antithyroid drugs for hyperthyroidism: a systematic review and meta-analysis
Source: Front Pharmacol. 2025 Feb 27;16:1530152. doi: 10.3389/fphar.2025.1530152 (PMC11903460; doi:10.3389/fphar.2025.1530152)

**Efficacy and safety of *Prunella vulgaris* L. combined with antithyroid drugs for hyperthyroidism: A systematic review and** **meta-analysis**

**Supplementary Files**

[Supplementary Table 1: PRISMA 2020 Checklist 1](#_Toc189258255)

[Supplementary Table 2 Systematic search detail (record number: 2024.7.18) 5](#_Toc189258256)

[Supplementary Table 3 Details of](#_Toc189258257) *[Prunella vulgaris](#_Toc189258257)* [L. used in the included studies 8](#_Toc189258257)

[Supplementary Table 4 Subgroup analysis of FT3, FT4, TSH, TRAb, TLTL, TNF-α and IL-6 10](#_Toc189258258)

[Supplementary Table 5 Details of adverse reactions 11](#_Toc189258259)

[Supplementary Table 6 Meta-regression 13](#_Toc189258260)

[Supplementary Table 7 Sensitivity analysis 15](#_Toc189258261)

[Supplementary Tabel 8 GRADE summary of outcomes for](#_Toc189258262) *[Prunella vulgaris](#_Toc189258262)* [L. combined with ATDs compared to ATDs for hyperthyroidism 18](#_Toc189258262)

[Supplementary Fig. 1 Subgroup analysis of FT3 21](#_Toc189258263)

[Supplementary Fig. 2 Subgroup analysis of FT4 23](#_Toc189258264)

[Supplementary Fig. 3 Subgroup analysis of TSH 25](#_Toc189258265)

[Supplementary Fig. 4 Subgroup analysis of TRAb 27](#_Toc189258266)

[Supplementary Fig. 5 Subgroup analysis of TLTL 28](#_Toc189258267)

[Supplementary Fig. 6 Subgroup analysis of TNF-α and IL-6 29](#_Toc189258268)

# Supplementary Table 1: PRISMA 2020 Checklist

| **Section and Topic** | **Item #** | **Checklist item** | **Location where item is reported** |
| --- | --- | --- | --- |
| **TITLE** | | |  |
| Title | 1 | Identify the report as a systematic review. |  |
| **ABSTRACT** | | |  |
| Abstract | 2 | See the PRISMA 2020 for Abstracts checklist. |  |
| **INTRODUCTION** | | |  |
| Rationale | 3 | Describe the rationale for the review in the context of existing knowledge. |  |
| Objectives | 4 | Provide an explicit statement of the objective(s) or question(s) the review addresses. |  |
| **METHODS** | | |  |
| Eligibility criteria | 5 | Specify the inclusion and exclusion criteria for the review and how studies were grouped for the syntheses. |  |
| Information sources | 6 | Specify all databases, registers, websites, organisations, reference lists and other sources searched or consulted to identify studies. Specify the date when each source was last searched or consulted. |  |
| Search strategy | 7 | Present the full search strategies for all databases, registers and websites, including any filters and limits used. | Supplementary Table 2 |
| Selection process | 8 | Specify the methods used to decide whether a study met the inclusion criteria of the review, including how many reviewers screened each record and each report retrieved, whether they worked independently, and if applicable, details of automation tools used in the process. |  |
| Data collection process | 9 | Specify the methods used to collect data from reports, including how many reviewers collected data from each report, whether they worked independently, any processes for obtaining or confirming data from study investigators, and if applicable, details of automation tools used in the process. |  |
| Data items | 10a | List and define all outcomes for which data were sought. Specify whether all results that were compatible with each outcome domain in each study were sought (e.g. for all measures, time points, analyses), and if not, the methods used to decide which results to collect. |  |
|  | 10b | List and define all other variables for which data were sought (e.g. participant and intervention characteristics, funding sources). Describe any assumptions made about any missing or unclear information. |  |
| Study risk of bias assessment | 11 | Specify the methods used to assess risk of bias in the included studies, including details of the tool(s) used, how many reviewers assessed each study and whether they worked independently, and if applicable, details of automation tools used in the process. |  |
| Effect measures | 12 | Specify for each outcome the effect measure(s) (e.g. risk ratio, mean difference) used in the synthesis or presentation of results. |  |
| Synthesis methods | 13a | Describe the processes used to decide which studies were eligible for each synthesis (e.g. tabulating the study intervention characteristics and comparing against the planned groups for each synthesis (item #5)). |  |
|  | 13b | Describe any methods used to tabulate or visually display results of individual studies and syntheses. |  |
|  | 13c | Describe any methods used to tabulate or visually display results of individual studies and syntheses. |  |
|  | 13d | Describe any methods used to synthesize results and provide a rationale for the choice(s). If meta-analysis was performed, describe the model(s), method(s) to identify the presence and extent of statistical heterogeneity, and software package(s) used. |  |
|  | 13e | Describe any methods used to explore possible causes of heterogeneity among study results (e.g. subgroup analysis, meta-regression). |  |
|  | 13f | Describe any sensitivity analyses conducted to assess robustness of the synthesized results. |  |
| Reporting bias assessment | 14 | Describe any methods used to assess risk of bias due to missing results in a synthesis (arising from reporting biases). |  |
| Certainty assessment | 15 | Describe any methods used to assess certainty (or confidence) in the body of evidence for an outcome. |  |
| **RESULTS** | | |  |
| Study selection | 16a | Describe the results of the search and selection process, from the number of records identified in the search to the number of studies included in the review, ideally using a flow diagram. | Figure 1 |
|  | 16b | Cite studies that might appear to meet the inclusion criteria, but which were excluded, and explain why they were excluded. |  |
| Study characteristics | 17 | Cite each included study and present its characteristics. | Table 1 |
| Risk of bias in studies | 18 | Present assessments of risk of bias for each included study. | Figure 2 |
| Results of individual studies | 19 | For all outcomes, present, for each study: (a) summary statistics for each group (where appropriate) and (b) an effect estimate and its precision (e.g. confidence/credible interval), ideally using structured tables or plots. | Figure 3-8. |
| Results of syntheses | 20a | For each synthesis, briefly summarize the characteristics and risk of bias among contributing studies. |  |
|  | 20b | Present results of all statistical syntheses conducted. If meta-analysis was done, present for each the summary estimate and its precision (e.g. confidence/credible interval) and measures of statistical heterogeneity. If comparing groups, describe the direction of the effect. | Figure 3-8 |
|  | 20c | Present results of all investigations of possible causes of heterogeneity among study results. | Supplementary Figure 1-6.  Supplementary Table 4. |
|  | 20d | Present results of all sensitivity analyses conducted to assess the robustness of the synthesized results. | Supplementary Table 7 |
| Reporting biases | 21 | Present assessments of risk of bias due to missing results (arising from reporting biases) for each synthesis assessed. | Figure 9 |
| Certainty of evidence | 22 | Present assessments of certainty (or confidence) in the body of evidence for each outcome assessed. | Supplementary Table 8 |
| **DISCUSSION** | | |  |
| Discussion | 23a | Provide a general interpretation of the results in the context of other evidence. |  |
|  | 23b | Discuss any limitations of the evidence included in the review. |  |
|  | 23c | Discuss any limitations of the review processes used. |  |
|  | 23d | Discuss implications of the results for practice, policy, and future research. |  |
| **OTHER INFORMATION** | | |  |
| Registration and protocol | 24a | Provide registration information for the review, including register name and registration number, or state that the review was not registered. |  |
|  | 24b | Indicate where the review protocol can be accessed, or state that a protocol was not prepared. |  |
|  | 24c | Describe and explain any amendments to information provided at registration or in the protocol. | None |
| Support | 25 | Describe sources of financial or non-financial support for the review, and the role of the funders or sponsors in the review. |  |
| Competing interests | 26 | Declare any competing interests of review authors. |  |
| Availability of data, code and other materials | 27 | Report which of the following are publicly available and where they can be found: template data collection forms; data extracted from included studies; data used for all analyses; analytic code; any other materials used in the review. |  |

# Supplementary Table 2 Systematic search detail (record number: 2024.7.18)

| **The search strategy for PubMed** | | |
| --- | --- | --- |
| **Sequence** | **Search terms** | **Hits** |
| #1 | Hyperthyroidism[MeSH Terms] | 46,808 |
| #2 | (((Hyperthyroid[Title/Abstract]) OR (Hyperthyroids[Title/Abstract])) OR (Primary Hyperthyroidism[Title/Abstract])) OR (Hyperthyroidism, Primary[Title/Abstract]) | 7,085 |
| #3 | Graves Disease[MeSH Terms] | 19,286 |
| #4 | ((((((((((((Disease, Graves[Title/Abstract]) OR (Basedow Disease[Title/Abstract])) OR (Disease, Basedow[Title/Abstract])) OR (Hyperthyroidism, Autoimmune[Title/Abstract])) OR (Exophthalmic Goiter[Title/Abstract])) OR (Exophthalmic Goiters[Title/Abstract])) OR (Goiters, Exophthalmic[Title/Abstract])) OR (Goiter, Exophthalmic[Title/Abstract])) OR (Graves' Disease[Title/Abstract])) OR (Disease, Graves'[Title/Abstract])) OR (Basedow's Disease[Title/Abstract])) OR (Basedows Disease[Title/Abstract])) OR (Disease, Basedow's[Title/Abstract]) | 15,737 |
| #5 | #1 OR #2 OR #3 OR #4 | 52,437 |
| #6 | Prunella[MeSH Terms] | 279 |
| #7 | (((((Prunellas[Title/Abstract]) OR (Prunella vulgaris[Title/Abstract])) OR (Prunella vulgari[Title/Abstract])) OR (vulgaris, Prunella[Title/Abstract])) OR (Xiakucao[Title/Abstract])) OR (Xiakucaos[Title/Abstract]) | 337 |
| #8 | #6 OR #7 | 417 |
| #9 | #5 AND #8 | 6 |
| **The search strategy for Web of science** | | |
| **Sequence** | **Search terms** | **Hits** |
| #1 | TS=(Hyperthyroidism) | 21,623 |
| #2 | (((AB=(Hyperthyroid)) OR AB=(Hyperthyroids)) OR AB=(Primary Hyperthyroidism)) OR AB=(Hyperthyroidism, Primary) | 5,989 |
| #3 | #1 OR #2 | 23,313 |
| #4 | TS=(Graves Disease) | 20,916 |
| #5 | ((((((((((((AB=(Disease, Graves)) OR AB=(Basedow Disease)) OR AB=(Disease, Basedow)) OR AB=(Hyperthyroidism, Autoimmune)) OR AB=(Exophthalmic Goiter)) OR AB=(Exophthalmic Goiters)) OR AB=(Goiters, Exophthalmic)) OR AB=(Goiter, Exophthalmic)) OR AB=(Graves' Disease)) OR AB=(Disease, Graves')) OR AB=(Basedow's Disease)) OR AB=(Basedow's Disease)) OR AB=(Disease, Basedow's) | 13,486 |
| #6 | #4 OR #5 | 21,563 |
| #7 | #3 OR #6 | 37,752 |
| #8 | TS=(Prunella) | 970 |
| #9 | (((((AB=(Prunellas)) OR AB=(Prunella vulgaris)) OR AB=(Prunella vulgari)) OR AB=(vulgaris, Prunella)) OR AB=(Xiakucao)) OR AB=(Xiakucaos) | 443 |
| #10 | #8 OR #9 | 971 |
| #11 | #7 AND #10 | 5 |
| **The search strategy for Cochrane Library** | | |
| **Sequence** | **Search terms** | **Hits** |
| #1 | MeSH descriptor:[Hyperthyroidism] explode all trees | 917 |
| #2 | (Hyperthyroid):ti,ab,kw OR (Hyperthyroids):ti,ab,kw OR (Primary Hyperthyroidism):ti,ab,kw OR (Hyperthyroidism, Primary):ti,ab,kw | 1,591 |
| #3 | MeSH descriptor: [Graves Disease] explode all trees | 591 |
| #4 | (Basedow Disease):ti,ab,kw OR (Graves' Disease):ti,ab,kw OR (Goiter, Exophthalmic):ti,ab,kw OR (Goiters, Exophthalmic):ti,ab,kw OR (Basedows Disease):ti,ab,kw | 1,074 |
| #5 | (Disease, Graves):ti,ab,kw OR (Disease, Basedow's):ti,ab,kw OR (Disease, Graves'):ti,ab,kw OR (Basedow's Disease):ti,ab,kw OR (Disease, Basedow):ti,ab,kw | 1,072 |
| #6 | (Exophthalmic Goiter):ti,ab,kw OR (Exophthalmic Goiters):ti,ab,kw OR (Hyperthyroidism, Autoimmune):ti,ab,kw | 143 |
| #7 | #1 OR #2 OR #3 OR #4 OR #5 OR #6 | 2,354 |
| #8 | MeSH descriptor: [Prunella] explode all trees | 5 |
| #9 | (Prunellas):ti,ab,kw OR (Xiakucaos):ti,ab,kw OR (Prunella vulgaris):ti,ab,kw OR (vulgaris, Prunella):ti,ab,kw OR (Prunella vulgari):ti,ab,kw | 30 |
| #10 | (Xiakucao):ti,ab,kw | 8 |
| #11 | #8 OR #9 OR #10 | 30 |
| #12 | #7 AND #11 | 4 |
| **The search strategy for EMBASE** | | |
| **Sequence** | **Search terms** | **Hits** |
| #1 | 'hyperthyroidism'/exp | 85,554 |
| #2 | 'hyperthyroid':ab,ti OR 'hyperthyroids':ab,ti OR 'primary hyperthyroidism':ab,ti OR 'hyperthyroidism, primary':ab,ti | 9,568 |
| #3 | 'graves disease'/exp | 27,543 |
| #4 | 'disease, graves':ab,ti OR 'basedow disease':ab,ti OR 'disease, basedow':ab,ti OR 'hyperthyroidism, autoimmune':ab,ti OR 'exophthalmic goiter':ab,ti OR 'exophthalmic goiters':ab,ti OR 'goiters, exophthalmic':ab,ti OR 'goiter, exophthalmic':ab,ti | 710 |
| #5 | #1 OR #2 OR #3 OR #4 | 87,161 |
| #6 | 'prunella'/exp | 506 |
| #7 | 'prunellas':ab,ti OR 'prunella vulgaris':ab,ti OR 'prunella vulgari':ab,ti OR 'vulgaris, prunella':ab,ti OR 'xiakucao':ab,ti OR 'xiakucaos':ab,ti | 445 |
| #8 | #6 OR #7 | 657 |
| #9 | #5 AND #8 | 13 |
| **The search strategy for CNKI** | | |
| **Sequence** | **Search terms** | **Hits** |
| #1 | (SU%=甲状腺功能亢进症) OR (SU%=甲状腺机能亢进症) OR (SU%=甲亢) OR (SU%=毒性弥漫性甲状腺肿) OR (SU%=Graves病) OR (SU%=格雷夫斯病) | 33,253 |
| #2 | (SU%=夏枯草口服液) OR (SU%=夏枯草胶囊) OR (SU%=夏枯草片) OR (SU%=夏枯草颗粒) OR (SU%=夏枯草膏) OR (SU%=夏枯草汤) OR (SU%=夏枯草) | - |
| #3 | #2 is retrieved in the result of #1 | 227 |
| **The search strategy for Wanfang Data** | | |
| **Sequence** | **Search terms** | **Hits** |
| #1 | (Theme=甲状腺功能亢进症) OR (Theme=甲状腺机能亢进症) OR (Theme=毒性弥漫性甲状腺肿) OR (Theme=Graves病) OR (Theme=格雷夫斯病) | 63,236 |
| #2 | (Theme=夏枯草口服液) OR (Theme=夏枯草胶囊) OR (Theme=夏枯草片) OR (Theme=夏枯草颗粒) OR (Theme=夏枯草膏) OR (Theme=夏枯草汤) OR (Theme=夏枯草) | 3,724 |
| #3 | #1 AND #2 | 103 |
| **The search strategy for SinoMed** | | |
| **Sequence** | **Search terms** | **Hits** |
| #1 | "甲状腺功能亢进症"[Common fields: Auto] OR "甲状腺机能亢进症"[Common fields: Auto] OR "毒性弥漫性甲状腺肿"[Common fields: Auto] OR "Graves病"[Common fields: Auto] OR "格雷夫斯病"[Common fields: Auto] | 28,865 |
| #2 | "夏枯草口服液"[Common fields: Auto] OR "夏枯草胶囊"[Common fields: Auto] OR "夏枯草片"[Common fields: Auto] OR "夏枯草颗粒"[Common fields: Auto] OR "夏枯草膏"[Common fields: Auto] OR "夏枯草汤"[Common fields: Auto] OR "夏枯草"[Common fields: Auto] | 2,230 |
| #3 | #1 AND #2 | 95 |
| The common fields consist of four search terms: Chinese title, abstract, keywords, and theme words. | | |
| **The search strategy for VIP** | | |
| **Sequence** | **Search terms** | **Hits** |
| #1 | M=甲状腺功能亢进症+甲状腺机能亢进症+毒性弥漫性甲状腺肿+Graves病+格雷夫斯病 | 11,715 |
| #2 | M=夏枯草口服液*+*夏枯草胶囊+夏枯草片+夏枯草颗粒+夏枯草膏+夏枯草汤+夏枯草 | 1,578 |
| #3 | #1 AND #2 | 31 |

# Supplementary Table 3 Details of *Prunella vulgaris* L. used in the included studies

| Preparation | Dosage Form | Specification | Ingredients | Approval Number | Manufacturer | Dose | Insurance Coverage | Indication | Contraindication |
| --- | --- | --- | --- | --- | --- | --- | --- | --- | --- |
| Xiakucao oral liquid | Oral liquid | 10ml | *Prunella vulgaris* L. | National medicine permission number  Z19990052 | Guiyang Xintian Pharmaceutical Co., Ltd. | 5~20ml/day | National Medical Insurance (2023) | (1) goiter (enlargement of thyroid gland)  (2) lymph node tuberculosis  (3) mammary hyperplasia | Unclear |
| Xiakucao capsule | Capsule | 0.35g | Total saponins of *Prunella vulgaris* L. | National medicine permission number  Z19991033 | Juxiechang (Beijing) Pharmaceutical Co., Ltd. | 1.40g/day | National Medical Insurance (2023) | (1) goiter (enlargement of thyroid gland)  (2) lymph node tuberculosis  (3) mammary hyperplasia | Unclear |
| Xiakucao granule | Granule | 9g | *Prunella vulgaris* L. | National medicine permission number  Z20050593 | Shandong Xianhe Pharmaceutical Co., Ltd. | 18g/day | National Medical Insurance (2023) | (1) goiter (enlargement of thyroid gland)  (2) lymph node tuberculosis  (3) mammary hyperplasia | Unclear |
| Xiakucao granule | Granule | 2g | *Prunella vulgaris* L. | National medicine permission number  Z20050519 | Jiangsu Chenpai Pharmaceutical Group Co., Ltd | 4g/day | National Medical Insurance (2023) | (1) goiter (enlargement of thyroid gland)  (2) lymph node tuberculosis  (3) mammary hyperplasia  (4) hypertension | Unclear |
| Xiakucao granule | Granule | 15g | *Prunella vulgaris* L. | - | Guangzhou Yifang Pharmaceutical Co., Ltd. | 30g/d | - | - | Unclear |

# Supplementary Table 4 Subgroup analysis of FT3, FT4, TSH, TRAb, TLTL, TNF-α and IL-6

| Outcomes | Subgroup | n | SMD/MD (95%CI) | *I^2^*(%) | *^1^P* | *^2^P* | *^3^P* |
| --- | --- | --- | --- | --- | --- | --- | --- |
| FT3 | Total | 17 | -0.98 [-1.39, -0.57] | 92 | <0.00001 | <0.00001 | - |
|  | Intervention duration  < 6 months | 11 | -0.95 [-1.44, -0.46] | 91 | 0.0002 | <0.00001 | 0.86 |
|  | Intervention duration  ≥ 6 months | 6 | -1.04 [-1.85, -0.22] | 94 | 0.01 | <0.00001 |  |
|  | Oral liquid | 7 | -0.60 [-1.13, -0.07] | 89 | 0.03 | <0.00001 | 0.28 |
|  | Capsule | 3 | -1.03 [-1.91, -0.15] | 88 | 0.02 | 0.0002 |  |
|  | Granule | 7 | -1.35 [-2.13, -0.56] | 94 | 0.0008 | <0.00001 |  |
|  | Sample size < 80 | 7 | -0.86 [-1.55, -0.18] | 91 | 0.01 | <0.00001 | 0.66 |
|  | Sample size ≥ 80 | 10 | -1.06 [-1.60, -0.52] | 93 | 0.0001 | <0.00001 |  |
| FT4 | Total | 17 | -0.82 [-1.16, -0.47] | 89 | <0.00001 | <0.00001 | - |
|  | Intervention duration  < 6 months | 11 | -0.79 [-1.21, -0.38] | 88 | 0.0002 | <0.00001 | 0.85 |
|  | Intervention duration  ≥ 6 months | 6 | -0.87 [-1.55, -0.19] | 92 | 0.01 | <0.00001 |  |
|  | Oral liquid | 7 | -0.52 [-1.01, -0.03] | 88 | 0.04 | <0.00001 | 0.33 |
|  | Capsule | 3 | -0.84 [-1.44, -0.24] | 77 | 0.006 | 0.01 |  |
|  | Granule | 7 | -1.11 [-1.72, -0.50] | 91 | 0.0004 | <0.00001 |  |
|  | Sample size < 80 | 7 | -0.74 [-1.32, -0.17] | 88 | 0.01 | <0.00001 | 0.73 |
|  | Sample size ≥ 80 | 10 | -0.87 [-1.33, -0.42] | 91 | 0.0002 | <0.00001 |  |
| TSH | Total | 17 | 0.71 [0.43, 0.99] | 82 | <0.00001 | <0.00001 | - |
|  | Intervention duration  < 6 months | 11 | 0.76 [0.39, 1.12] | 84 | 0.0001 | <0.00001 | 0.68 |
|  | Intervention duration  ≥ 6 months | 6 | 0.63 [0.18, 1.09] | 84 | 0.007 | <0.00001 |  |
|  | Oral liquid | 7 | 0.51 [0.09, 0.92] | 83 | 0.02 | 0.00001 | 0.08 |
|  | Capsule | 3 | 0.44 [0.16, 0.72] | 0 | 0.002 | 0.69 |  |
|  | Granule | 7 | 1.08 [0.59, 1.57] | 85 | <0.0001 | <0.00001 |  |
|  | Sample size < 80 | 7 | 0.71 [0.05, 1.37] | 89 | 0.03 | <0.00001 | 1.00 |
|  | Sample size ≥ 80 | 10 | 0.71 [0.42, 1.00] | 78 | <0.00001 | <0.00001 |  |
| TRAb | Total | 4 | -1.11 [-1.52, -0.71] | 65 | <0.00001 | 0.04 | - |
|  | Intervention duration  < 6 months | 3 | -1.00 [-1.42, -0.57] | 63 | <0.00001 | 0.07 | 0.13 |
|  | Intervention duration  ≥ 6 months | 1 | -1.57 [-2.17, -0.96] | - | <0.00001 | - |  |
|  | Oral liquid | 3 | -1.26 [-1.69, -0.83] | 56 | <0.00001 | 0.10 | 0.07 |
|  | Granule | 1 | -0.68 [-1.14, -0.22] | - | 0.004 | - |  |
|  | Sample size < 80 | 2 | -1.10 [-1.97, -0.23] | 81 | 0.01 | 0.02 | 0.93 |
|  | Sample size ≥ 80 | 2 | -1.15 [-1.68, -0.61] | 66 | <0.0001 | 0.09 |  |
| TLTL | Total | 5 | -0.22 [-0.33, -0.10] | 74 | 0.0003 | 0.004 | - |
|  | Intervention duration  < 6 months | 1 | -0.45 [-0.61, -0.29] | - | <0.00001 | - | 0.002 |
|  | Intervention duration  ≥ 6 months | 4 | -0.17 [-0.25, -0.08] | 36 | <0.0001 | 0.20 |  |
|  | Sample size < 80 | 2 | -0.21 [-0.32, -0.11] | 0 | <0.0001 | 0.78 | 0.96 |
|  | Sample size ≥ 80 | 3 | -0.22 [-0.43, -0.01] | 87 | 0.04 | 0.0005 |  |
| TNF-α | Total | 3 | -2.05 [-2.85, -1.25] | 84 | <0.00001 | 0.002 | - |
|  | Sample size < 80 | 1 | -1.26 [-1.80, -0.73] | - | <0.00001 | - | 0.0005 |
|  | Sample size ≥ 80 | 2 | -2.45 [-2.86, -2.04] | 0 | <0.00001 | 0.82 |  |
| IL-6 | Total | 3 | -0.29 [-0.83, 0.26] | 77 | 0.30 | 0.01 | - |
|  | Sample size < 80 | 1 | -0.89 [-1.40, -0.38] | - | 0.0006 | - | 0.003 |
|  | Sample size ≥ 80 | 2 | -0.01 [-0.31, 0.30] | 0 | 0.96 | 1.00 |  |

^1^p values for effect size; ^2^p values for heterogeneity; ^3^p values for between subgroup.

# Supplementary Table 5 Details of adverse reactions

| Study (year) | Group | Sample size | Adverse event | Adverse reaction |
| --- | --- | --- | --- | --- |
| Chai et al., 2020^[1]^ | E | 50 | 3 | Leucopenia in 1 patient, liver dysfunction in 1 patient, secondary hypothyroidism in 1 patient |
|  | C | 50 | 18 | Leucopenia in 4 patients, liver dysfunction in 5 patients, mild rash in 4 patients, secondary hypothyroidism in 5 patients |
| Chen., 2023^[2]^ | E | 28 | 2 | Nausea and vomiting in 1 patient, 1 patient experienced burning sensation in mouth |
|  | C | 28 | 3 | Nausea and vomiting in 1 patient, diarrhea in 1 patient, 1 patient experienced burning sensation in mouth |
| Guo, 2018^[3]^ | E | 30 | 1 | Mild liver dysfunction in 1 patient |
|  | C | 32 | 5 | Mild liver dysfunction in 3 patients, leucopenia in 2 patients |
| Jia, 2022^[4]^ | E | 32 | 4 | Rash in 1 patient, palpitation in 1 patient, liver dysfunction in 1 patient, gastrointestinal discomfort in 1 patient |
|  | C | 32 | 6 | Rash in 2 patients, palpitation in 1 patient, headache in 1 patient, leucopenia in 1 patient, liver dysfunction in 1 patient |
| Jiang et al., 2022^[5]^ | E | 40 | 5 | liver dysfunction in 1 patient, nausea and vomiting in 3 patients, rash in 1 patient |
|  | C | 40 | 14 | liver dysfunction in 3 patients, nausea and vomiting in 7 patients, rash in 4 patients |
| Kan and Yi, 2020^[6]^ | E | 43 | 3 | Leucopenia in 1 patient, liver dysfunction in 1 patient, drug rash in 1 patient |
|  | C | 43 | 10 | Leucopenia in 5 patients, liver dysfunction in 2 patients, drug rash in 3 patients |
| Wu, 2012^[7]^ | E | 60 | 0 | None |
|  | C | 60 | 0 | None |
| Yang, 2023^[8]^ | E | 34 | 3 | Liver dysfunction in 2 patients, leucopenia in 1 patient |
|  | C | 32 | 9 | Liver dysfunction in 5 patients, leucopenia in 2 patients, rash in 2 patients |
| Yin, 2016^[9]^ | E | 49 | 7 | Headache and dizziness in 2 patients, anxiety and depression in 3 patients, rash in 2 patients |
|  | C | 49 | 16 | Headache and dizziness in 6 patients, anxiety and depression in 6 patients, rash in 4 patients |
| Zheng, 2021^[10]^ | E | 49 | 4 | Nausea and vomiting in 2 patients, liver dysfunction in 1 patient, rash in 1 patient |
|  | C | 49 | 12 | Nausea and vomiting in 3 patients, headache in 2 patients, liver dysfunction in 5 patients, rash in 2 patients |

E: Experimental group; C: control group.

**References**

[1] Chai, L. C., Wang, J., Wei, Y., and Liu, C. N. (2020). Clinical observation of Prunella vulgaris oral liquid combined with metimidazole in the treatment of syndrome of hyperactivity of heart and liver fire of Graves' disease. *Chin J Hosp Pharm.* 40 (11), 1246-1251.

[2] Chen, X. (2023). Clinical observation on the treatment of toxic diffuse goiter with Xiakucao oral liquid combined with methimazole tablets. *Journal of Practical Traditional Chinese Medicine.* 39 (11), 2162-2164.

[3] Guo, S. S. Clinical observations on the acue phase of Graves'disease (the excess of liver-fire syndrome) treated by Xiakucao capsule combined with Methidathion [D]: Henan University of Chinese Medicine, China, (2018).

[4] Jia, M. J., and Wei, W. (2022). Observation of the therapeutic effect of Xiakucao capsule combined with methimazole tablet in the treatment of hyperthyroidism. *Modern Medicine and Health Research.* 6 (9), 1-4.

[5] Jiang, L. Y., Chen, K., Pan, H. B., and Xia, L. (2022). Clinical observation on the treatment of hyperthyroidism with Xiakucao granule combined with methimazole tablet. *Journal of Henan Medical College for Staff and Workers.* 34 (4), 435-438.

[6] Kan, G. D., and Yi, F. L. (2020). Observation of the therapeutic effect of Xiakucao granules combined with methimazole tablets on hyperthyroidism and their effects on thyroid hormones, bone metabolism indicators, and immune related cytokines in patients. *Hebei Journal of Traditional Chinese Medicine.* 42 (6), 892-895+901.

[7] Wu, S. B. (2012). Clinical effect of Xiakucao oral liquid on the Graves disease. *Chin Tradit Pat Med.* 34 (1), 10-12.

[8] Yang, J. X. Effect of Xiakucao granules on inflammation and oxidative stress indexes in Graves' disease [D]: Guangzhou University of Chinese Medicine, China, (2023).

[9] Yin, Z. Y. (2016). Clinical observation of Xiakucao granules combined with Thiamazole tablets in treatment of diffuse goiter with hyperthyroidism. *Drugs Clin.* 31 (1), 70-74.

[10] Zheng, Y. L. (2021). Effects of Xiakucao granules combined with Methimazole in treatment of primary hyperthyroidism. *Medical Journal of Chinese People's Health.* 33 (19), 91-93.

# Supplementary Table 6 Meta-regression

| Outcome | Model | N | *τ^2^* | *I^2^* | *R^2^* | Coef. | Std. Err. | t | *P* | 95% Conf. Interval |
| --- | --- | --- | --- | --- | --- | --- | --- | --- | --- | --- |
| FT3 | Univariate | | | | | | |  |  |  |
|  | Intervention duration | 17 | 0.959 | 92.58% | -7.27% | -0.079 | 0.519 | -0.15 | 0.881 | (-1.185, 1.027) |
|  | Type of PVL preparations | 17 | 0.834 | 91.71% | 6.76% | -0.369 | 0.256 | -1.44 | 0.170 | (-0.914, 0.177) |
|  | Sample size | 17 | 0.951 | 92.58% | -6.34% | -0.199 | 0.503 | -0.40 | 0.698 | (-1.270, 0.873) |
|  | All covariates | | | | | | |  |  |  |
|  |  | 17 | 0.885 | 92.14% | 1.06% |  |  |  |  |  |
|  | Intervention duration | 17 |  |  |  | -0.654 | 0.611 | -1.07 | 0.304 | (-1.974, 0.666) |
|  | Type of PVL preparations | 17 |  |  |  | -0.556 | 0.322 | -1.73 | 0.107 | (-1.251, 0.139) |
|  | Sample size | 17 |  |  |  | 0.007 | 0.503 | 0.01 | 0.990 | (-1.081, 1.094) |
| FT4 | Univariate | | | | | | |  |  |  |
|  | Intervention duration | 17 | 0.604 | 89.82% | -7.55% | -0.069 | 0.419 | -0.16 | 0.872 | (-0.961, 0.823) |
|  | Type of PVL preparations | 17 | 0.523 | 88.24% | 6.96% | -0.291 | -0.207 | -1.41 | 0.179 | (-0.732, 0.149) |
|  | Sample size | 17 | 0.601 | 89.81% | -6.92% | -0.132 | 0.407 | -0.33 | 0.749 | (-0.999, 0.734) |
|  | All covariates | | | | | | |  |  |  |
|  |  | 17 | 0.558 | 88.87% | 0.66% |  |  |  |  |  |
|  | Intervention duration | 17 |  |  |  | -0.529 | 0.494 | -1.07 | 0.304 | (-1.597, 0.538) |
|  | Type of PVL preparations | 17 |  |  |  | -0.444 | 0.260 | -1.71 | 0.111 | (-1.006, 0.117) |
|  | Sample size | 17 |  |  |  | 0.032 | 0.408 | 0.08 | 0.939 | (-0.849, 0.913) |
| TSH | Univariate | | | | | | |  |  |  |
|  | Intervention duration | 16 | 0.331 | 83.81% | -7.67% | -0.125 | 0.326 | -0.38 | 0.708 | (-0.824, 0.574) |
|  | Type of PVL preparations | 16 | 0.262 | 80.92% | 14.64% | 0.279 | 0.159 | 1.76 | 0.101 | (-0.062, 0.621) |
|  | Sample size | 16 | 0.334 | 83.99% | -8.85% | 0.010 | 0.331 | 0.03 | 0.976 | (-0.699, 0.720) |
|  | All covariates | | | | | | |  |  |  |
|  |  | 16 | 0.304 | 83.29% | 0.99% |  |  |  |  |  |
|  | Intervention duration | 16 |  |  |  | 0.238 | 0.375 | 0.63 | 0.539 | (-0.580, 1.055) |
|  | Type of PVL preparations | 16 |  |  |  | 0.366 | 0.207 | 1.77 | 0.102 | (-0.085, 0.817) |
|  | Sample size | 16 |  |  |  | 0.164 | 0.334 | -0.49 | 0.632 | (-0.892, 0.564) |

# Supplementary Table 7 Sensitivity analysis

| Outcome | Omitted Study | Data with study removed MD/SMD/RR (95% CI) | *P* | *I^2^* |
| --- | --- | --- | --- | --- |
| FT3 | Chai 2020 | -0.94 [-1.36, -0.51] | <0.0001 | 92% |
|  | Chen 2023 | -0.93 [-1.35, -0.51] | <0.0001 | 92% |
|  | Guo 2018 | -1.00 [-1.44, -0.57] | <0.00001 | 93% |
|  | Jia 2022 | -0.92 [-1.33, -0.50] | <0.0001 | 92% |
|  | Jiang 2022 | -0.84 [-1.21, -0.47] | <0.00001 | 90% |
|  | Jin 2023 | -0.93 [-1.36, -0.51] | < 0.0001 | 92% |
|  | Kan 2020 | -1.00 [-1.44, -0.55] | < 0.0001 | 93% |
|  | Liang 2010 | -1.04 [-1.47, -0.61] | < 0.00001 | 92% |
|  | Lu 2018 | -1.01 [-1.45, -0.57] | < 0.00001 | 93% |
|  | Wang 2017 | -0.98 [-1.42, -0.54] | < 0.0001 | 93% |
|  | Wu 2012 | -1.04 [-1.47, -0.61] | < 0.00001 | 92% |
|  | Xie 2015 | -1.02 [-1.46, -0.58] | < 0.00001 | 92% |
|  | Yang 2007 | -1.04 [-1.47, -0.61] | < 0.00001 | 92% |
|  | Yang 2023 | -1.05 [-1.47, -0.62] | < 0.00001 | 92% |
|  | Yin 2007 | -1.02 [-1.46, -0.58] | < 0.00001 | 92% |
|  | Zheng 2021 | -0.89 [-1.28, -0.49] | < 0.0001 | 91% |
|  | Zou 2016 | -1.02 [-1.46, -0.58] | < 0.00001 | 92% |
| FT4 | Chai 2020 | -0.79 [-1.14, -0.43] | < 0.0001 | 89% |
|  | Chen 2023 | -0.76 [-1.10, -0.41] | <0.0001 | 89% |
|  | Guo 2018 | -0.83 [-1.20, -0.47] | <0.00001 | 90% |
|  | Jia 2022 | -0.78 [-1.13, -0.42] | <0.0001 | 89% |
|  | Jiang 2022 | -0.70 [-1.01, -0.40] | <0.00001 | 86% |
|  | Jin 2023 | -0.79 [-1.15, -0.43] | <0.0001 | 89% |
|  | Kan 2020 | -0.82 [-1.19, -0.45] | <0.0001 | 90% |
|  | Liang 2010 | -0.87 [-1.23, -0.52] | <0.00001 | 89% |
|  | Lu 2018 | -0.84 [-1.21, -0.48] | <0.00001 | 90% |
|  | Wang 2017 | -0.82 [-1.19, -0.45] | <0.0001 | 90% |
|  | Wu 2012 | -0.87 [-1.23, -0.52] | <0.00001 | 89% |
|  | Xie 2015 | -0.86 [-1.22, -0.49] | <0.00001 | 89% |
|  | Yang 2007 | -0.87 [-1.22, -0.51] | <0.00001 | 89% |
|  | Yang 2023 | -0.87 [-1.23, -0.52] | <0.00001 | 89% |
|  | Yin 2007 | -0.85 [-1.22, -0.48] | <0.00001 | 90% |
|  | Zheng 2021 | -0.76 [-1.10, -0.42] | <0.0001 | 88% |
|  | Zou 2016 | -0.86 [-1.22, -0.49] | <0.00001 | 89% |
| TSH | Chai 2020 | 0.66 [0.38, 0.94] | <0.00001 | 82% |
|  | Chen 2023 | 0.67 [0.39, 0.96] | <0.00001 | 83% |
|  | Guo 2018 | 0.74 [0.45, 1.03] | <0.00001 | 84% |
|  | Jia 2022 | 0.72 [0.43, 1.02] | <0.00001 | 84% |
|  | Jiang 2022 | 0.65 [0.38, 0.92] | <0.00001 | 81% |
|  | Jin 2023 | 0.62 [0.38, 0.85] | <0.00001 | 76% |
|  | Kan 2020 | 0.69 [0.39, 0.98] | <0.00001 | 83% |
|  | Liang 2010 | 0.75 [0.47, 1.04] | <0.00001 | 83% |
|  | Lu 2018 | 0.72 [0.42, 1.02] | <0.00001 | 84% |
|  | Wang 2017 | 0.72 [0.42, 1.01] | <0.00001 | 84% |
|  | Wu 2012 | 0.74 [0.44, 1.03] | <0.00001 | 83% |
|  | Xie 2015 | 0.75 [0.47, 1.04] | <0.00001 | 82% |
|  | Yang 2007 | 0.75 [0.46, 1.03] | <0.00001 | 83% |
|  | Yang 2023 | 0.71 [0.43, 0.99] | <0.00001 | 83% |
|  | Yin 2007 | 0.73 [0.43, 1.02] | <0.00001 | 84% |
|  | Zheng 2021 | 0.71 [0.41, 1.01] | <0.0001 | 84% |
|  | Zou 2016 | 0.72 [0.43, 1.02] | <0.00001 | 84% |
| TRAb | Chai 2020 | -1.21 [-1.76, -0.65] | <0.0001 | 73% |
|  | Chen 2023 | -1.00 [-1.42, -0.57] | <0.00001 | 63% |
|  | Jin 2023 | -1.26 [-1.69, -0.83] | <0.00001 | 56% |
|  | Zou 2016 | -1.00 [-1.47, -0.54] | <0.0001 | 63% |
| WLTL | Chen 2023 | -0.21 [-0.27, -0.15] | <0.00001 | 0% |
|  | Wu 2012 | -0.21 [-0.27, -0.15] | <0.00001 | 0% |
|  | Xie 2015 | -0.24 [-0.30, -0.17] | <0.00001 | 0% |
|  | Yang 2007 | -0.23 [-0.29, -0.17] | <0.00001 | 0% |
|  | Zou 2016 | -0.22 [-0.28, -0.16] | <0.00001 | 0% |
| TLTL | Chen 2023 | -0.22 [-0.37, -0.07] | 0.004 | 80% |
|  | Wu 2012 | -0.23 [-0.39, -0.06] | 0.008 | 80% |
|  | Xie 2015 | -0.26 [-0.37, -0.15] | <0.00001 | 64% |
|  | Yang 2007 | -0.21 [-0.36,- 0.07] | 0.005 | 80% |
|  | Zou 2016 | -0.17 [-0.25, -0.08] | <0.0001 | 36% |
| LLTL | Chen 2023 | -0.61 [-0.78, -0.44] | <0.00001 | 0% |
|  | Wu 2012 | -0.60 [-0.79, -0.41] | <0.00001 | 0% |
|  | Xie 2015 | -0.71 [-0.90, -0.52] | <0.00001 | 0% |
|  | Yang 2007 | -0.61 [-0.78, -0.44] | <0.00001 | 0% |
| TIT | Chen 2023 | -0.12 [-0.15, -0.09] | <0.00001 | 21% |
|  | Wu 2012 | -0.12 [-0.15, -0.09] | <0.00001 | 21% |
|  | Xie 2015 | -0.14 [-0.16, -0.11] | <0.00001 | 0% |
|  | Yang 2007 | -0.13 [-0.16, -0.10] | <0.00001 | 33% |
| WRTL | Chen 2023 | -0.20 [-0.26, -0.14] | <0.00001 | 42% |
|  | Wu 2012 | -0.19 [-0.25, -0.12] | <0.00001 | 27% |
|  | Xie 2015 | -0.24 [-0.30, -0.17] | <0.00001 | 0% |
|  | Yang 2007 | -0.20 [-0.26, -0.14] | <0.00001 | 43% |
|  | Zou 2016 | -0.23 [-0.29, -0.16] | <0.00001 | 36% |
| TRTL | Chen 2023 | -0.28 [-0.33, -0.22] | <0.00001 | 51% |
|  | Wu 2012 | -0.30 [-0.36, -0.24] | <0.00001 | 29% |
|  | Xie 2015 | -0.27 [-0.32, -0.21] | <0.00001 | 52% |
|  | Yang 2007 | -0.28 [-0.33, -0.22] | <0.00001 | 50% |
|  | Zou 2016 | -0.24 [-0.30, -0.18] | <0.00001 | 0% |
| LRTL | Chen 2023 | -0.44 [-0.62, -0.27] | <0.00001 | 0% |
|  | Wu 2012 | -0.45 [-0.66, -0.25] | <0.0001 | 0% |
|  | Xie 2015 | -0.45 [-0.64, -0.27] | <0.00001 | 0% |
|  | Yang 2007 | -0.43 [-0.61, -0.25] | <0.00001 | 0% |
| TNF-α | Kan 2020 | -1.88 [-3.09, -0.66] | 0.002 | 89% |
|  | Wang 2017 | -1.83 [-2.95, -0.71] | 0.001 | 88% |
|  | Yang 2023 | -2.45 [-2.86, -2.04] | <0.00001 | 0% |
| IL-6 | Kan 2020 | -0.44 [-1.31, 0.43] | 0.32 | 85% |
|  | Wang 2017 | -0.44 [-1.30, 0.43] | 0.32 | 85% |
|  | Yang 2023 | -0.01 [-0.31, 0.30] | 0.96 | 0% |
| IL-10 | Kan 2020 | 1.73 [1.20, 2.26] | <0.00001 | - |
|  | Wang 2017 | 1.72 [1.18, 2.26] | <0.00001 | - |
| IFN-γ | Kan 2020 | -0.12 [-1.87, 1.63] | 0.89 | - |
|  | Wang 2017 | -0.09 [-1.80, 1.62] | 0.92 | - |
| Relapse rate | Chai 2020 | 0.48 [0.13, 1.72] | 0.26 | 49% |
|  | Chen 2023 | 0.43 [0.09, 1.93] | 0.27 | 59% |
|  | Wu 2012 | 0.15 [0.04, 0.66] | 0.01 | 0% |
| Adverse event | Chai 2020 | 0.39 [0.26, 0.57] | <0.00001 | 0% |
|  | Chen 2023 | 0.33 [0.23, 0.49] | <0.00001 | 0% |
|  | Guo 2018 | 0.35 [0.24, 0.51] | <0.00001 | 0% |
|  | Jia 2022 | 0.32 [0.22, 0.48] | <0.00001 | 0% |
|  | Jiang 2022 | 0.34 [0.23, 0.51] | <0.00001 | 0% |
|  | Kan 2020 | 0.35 [0.24, 0.52] | <0.00001 | 0% |
|  | Wu 2012 | 0.34 [0.24, 0.50] | <0.00001 | 0% |
|  | Yang 2023 | 0.35 [0.23, 0.51] | <0.00001 | 0% |
|  | Yin 2007 | 0.32 [0.21, 0.49] | <0.00001 | 0% |
|  | Zheng 2021 | 0.34 [0.23, 0.51] | <0.00001 | 0% |

p values for effect size.

# Supplementary Tabel 8 GRADE summary of outcomes for *Prunella vulgaris* L. combined with ATDs compared to ATDs for hyperthyroidism

| Outcomes | Risk of bias | Inconsistency | Indirectness | Imprecision | Other  considerations | No. of patients (studies) | Relative  (95% CI) | Absolute  (95% CI) | Certainty of the evidence | Importance |
| --- | --- | --- | --- | --- | --- | --- | --- | --- | --- | --- |
| FT3 | serious^a^ | serious^b^ | not serious | not serious | publication bias strongly suspected^c^ | 1360(17) | - | SMD 0.98 lower(1.39 lower to 0.57 lower) | ⨁◯◯◯Very low | IMPORTANT |
| FT4 | serious^a^ | serious^b^ | not serious | not serious | publication bias strongly suspected^c^ | 1360(17) | - | SMD 0.82 lower(1.16 lower to 0.47 lower) | ⨁◯◯◯ Very low | IMPORTANT |
| TSH | serious^a^ | serious^b^ | not serious | not serious | none | 1360(17) | - | SMD 0.71 higher(0.43 higher to 0.99 higher) | ⨁⨁◯◯ Low | IMPORTANT |
| TRAb | serious^a^ | serious^b^ | not serious | serious^d^ | publication bias strongly suspected^c^ | 324(4) | - | SMD 1.11 lower(1.52 lower to 0.71 lower) | ⨁◯◯◯Very low | IMPORTANT |
| WLTL | serious^a^ | not serious | not serious | serious^d^ | publication bias strongly suspected^c^ | 408(5) | - | MD 0.22 lower(0.27 lower to 0.17 lower) | ⨁◯◯◯Very low | NOT  IMPORTANT |
| TLTL | serious^a^ | serious^b^ | not serious | serious^d^ | publication bias strongly suspected^c^ | 408(5) | - | MD 0.22 lower(0.33 lower to 0.10 lower) | ⨁◯◯◯Very low | NOT  IMPORTANT |
| LLTL | serious^a^ | not serious | not serious | serious^d^ | publication bias strongly suspected^c^ | 318(4) | - | MD 0.63 lower(0.79 lower to 0.47 lower) | ⨁◯◯◯Very low | NOT  IMPORTANT |
| TIT | serious^a^ | not serious | not serious | serious^d^ | publication bias strongly suspected^c^ | 318(4) | - | MD 0.13 lower(0.15 lower to 0.10 lower) | ⨁◯◯◯Very low | NOT  IMPORTANT |
| WRTL | serious^a^ | not serious | not serious | serious^d^ | publication bias strongly suspected^c^ | 408(5) | - | MD 0.21 lower(0.26 lower to 0.16 lower) | ⨁◯◯◯Very low | NOT IMPORTANT |
| TRTL | serious^a^ | not serious | not serious | serious^d^ | publication bias strongly suspected^c^ | 408(5) | - | MD 0.27 lower(0.32 lower to 0.22 lower) | ⨁◯◯◯Very low | NOT IMPORTANT |
| LRTL | serious^a^ | not serious | not serious | serious^d^ | publication bias strongly suspected^c^ | 318(4) | - | MD 0.45 lower(0.61 lower to 0.28 lower) | ⨁◯◯◯Very low | NOT IMPORTANT |
| TNF-α | serious^a^ | serious^b^ | not serious | serious^d^ | publication bias strongly suspected^c^ | 232(3) | - | SMD 2.05 lower(2.85 lower to 1.25 lower) | ⨁◯◯◯Very low | NOT IMPORTANT |
| IL-6 | serious^a^ | serious^b^ | not serious | serious^d^ | publication bias strongly suspected^c^ | 232(3) | - | SMD 0.29 lower(0.83 lower to 0.26 higher) | ⨁◯◯◯Very low | NOT IMPORTANT |
| IL-10 | serious^a^ | not serious | not serious | serious^d^ | publication bias strongly suspected^c^ | 166(2) | - | MD 1.73 higher(1.35 higher to 2.10 higher) | ⨁◯◯◯Very low | NOT IMPORTANT |
| IFN-γ | serious^a^ | not serious | not serious | serious^d^ | publication bias strongly suspected^c^ | 166(2) | - | MD 0.10 lower(1.33 lower to 1.12 higher) | ⨁◯◯◯Very low | NOT IMPORTANT |
| Relapse rate | serious^a^ | serious^b^ | not serious | serious^d^ | publication bias strongly suspected^c^ | 276(3) | RR 0.35  (0.10 to 1.24) | 231 fewer per 1000 (from 320 fewer to 85 more) | ⨁◯◯◯Very low | NOT IMPORTANT |
| Adverse event | serious^a^ | not serious | not serious | not serious | none | 770(10) | RR 0.34  (0.24 to 0.50) | 159 fewer per 1000 (from 184 fewer to 121 fewer) | ⨁⨁⨁◯Moderate | NOT IMPORTANT |

a. The risk of bias is decreased by one level: poor methodological quality, such as random sequence generation, allocation concealment, blinding, selective reporting, and others. As shown in Figure 2.

b. The inconsistency is reduced by one level: I^2^≥ 50% for heterogeneity.

c. Egger test or a funnel plot indicates potential publication bias.

d. The inaccuracy is decreased by one level: Small sample sizes (< 500).

# **Supplementary Fig. 1 Subgroup analysis of FT3**

(A) Intervention duration (T < 6months, T ≥ 6 months); (B) PVL preparations (oral liquid, capsule, granule) (C) Sample size (sample size < 80, sample size ≥ 80).


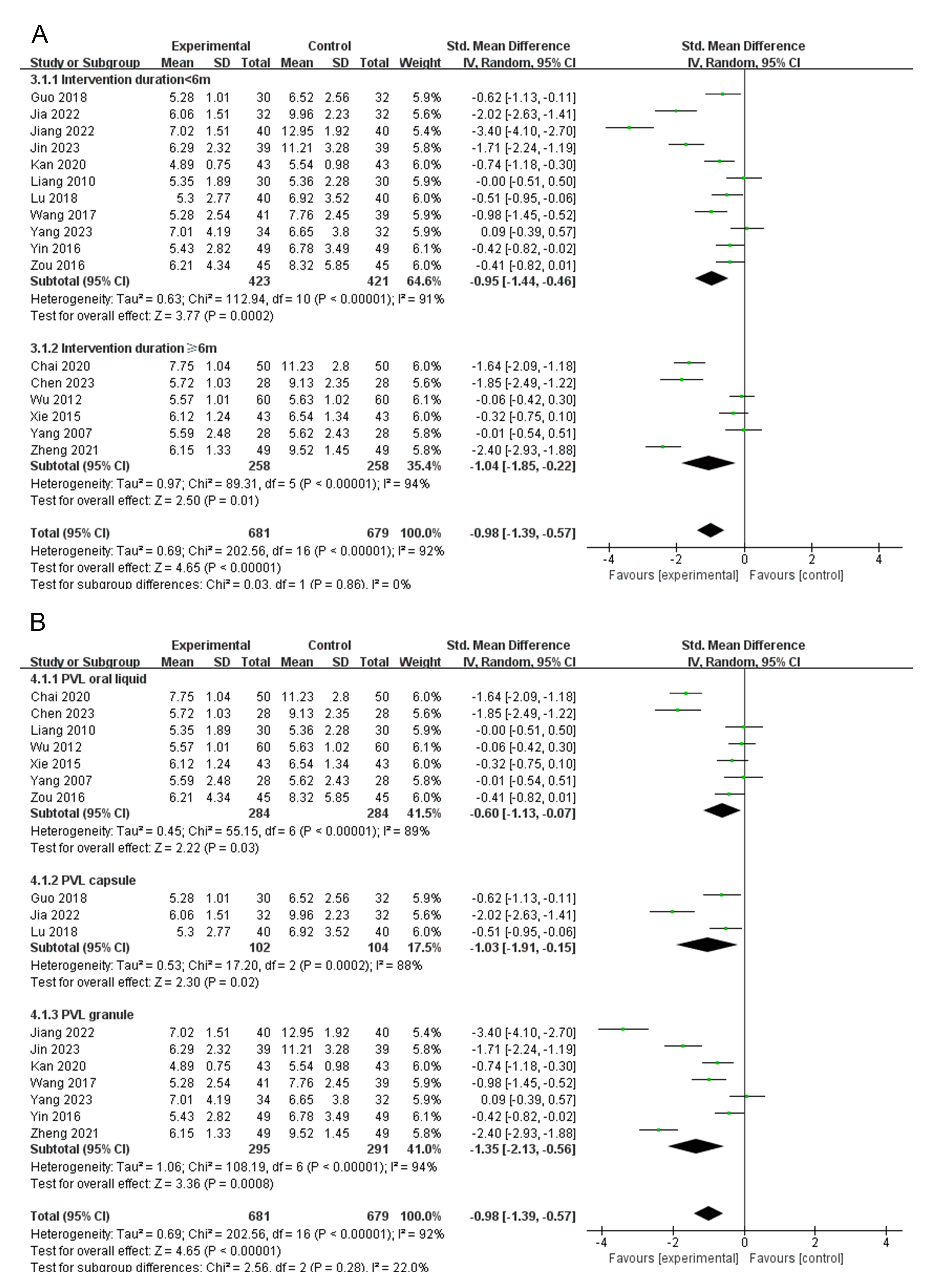


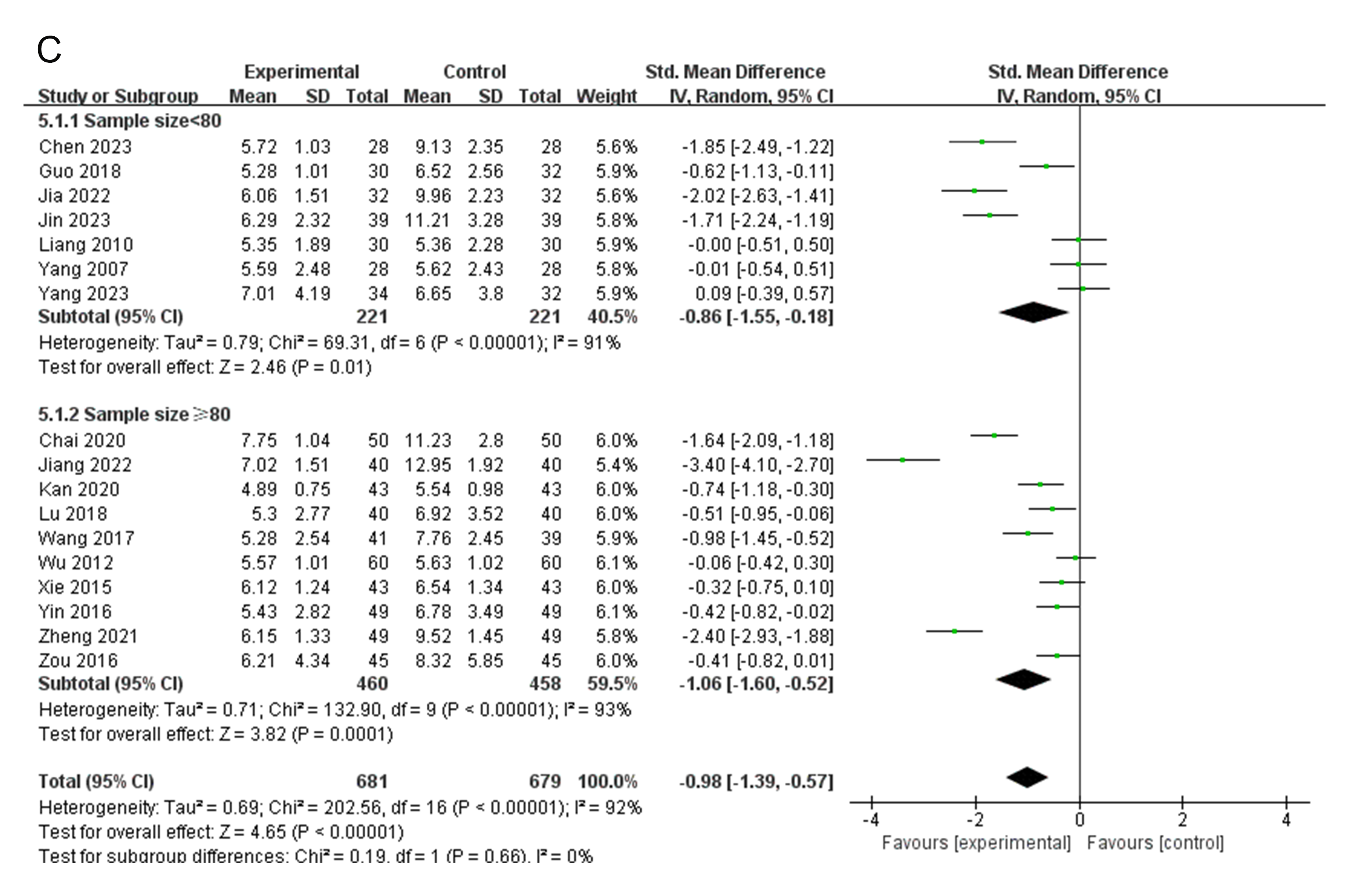


# **Supplementary Fig. 2 Subgroup analysis of FT4**

(A)Intervention duration (T < 6months, T ≥ 6 months); (B) PVL preparations (oral liquid, capsule, granule) (C) Sample size (sample size < 80, sample size ≥ 80).


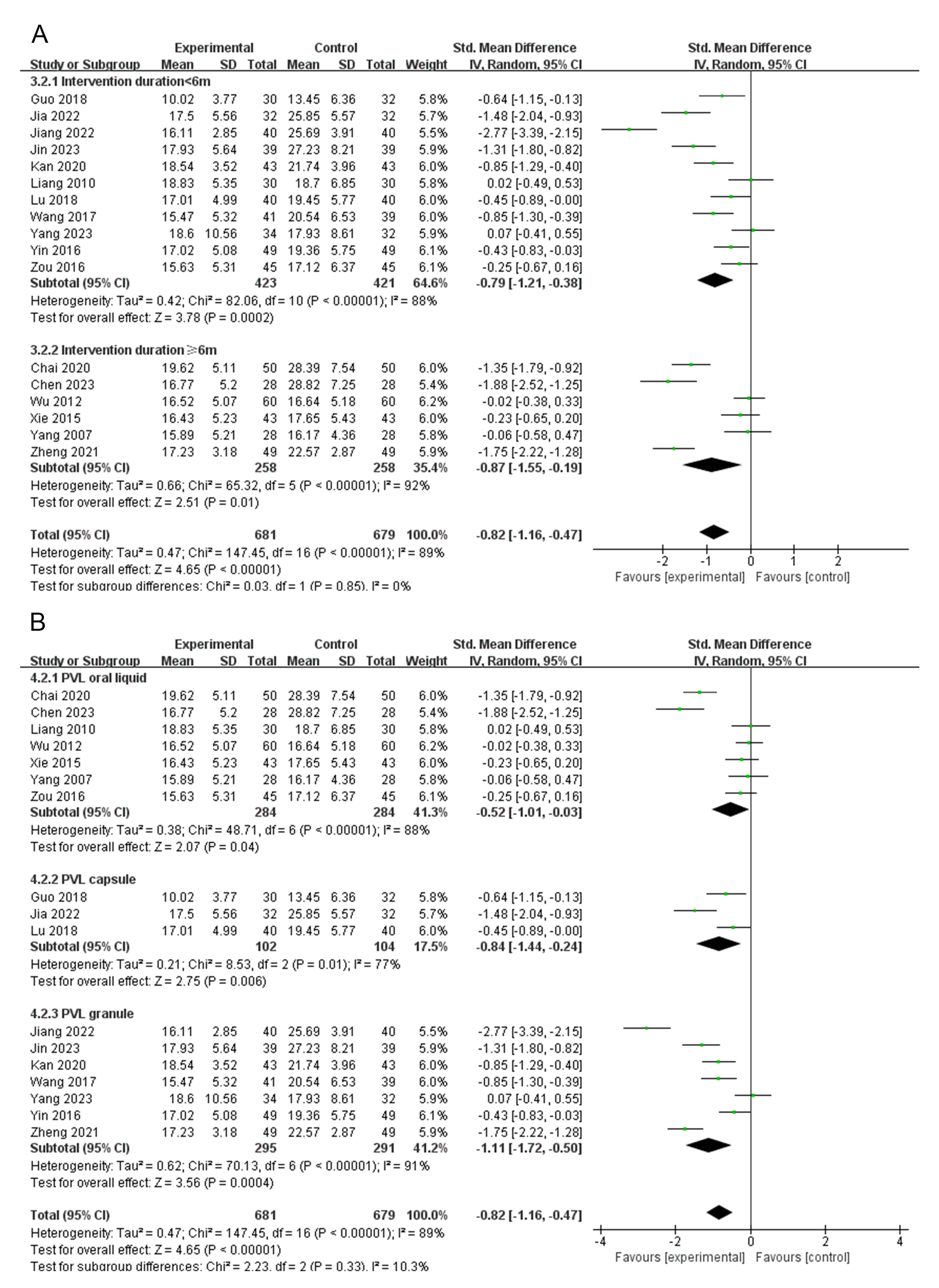


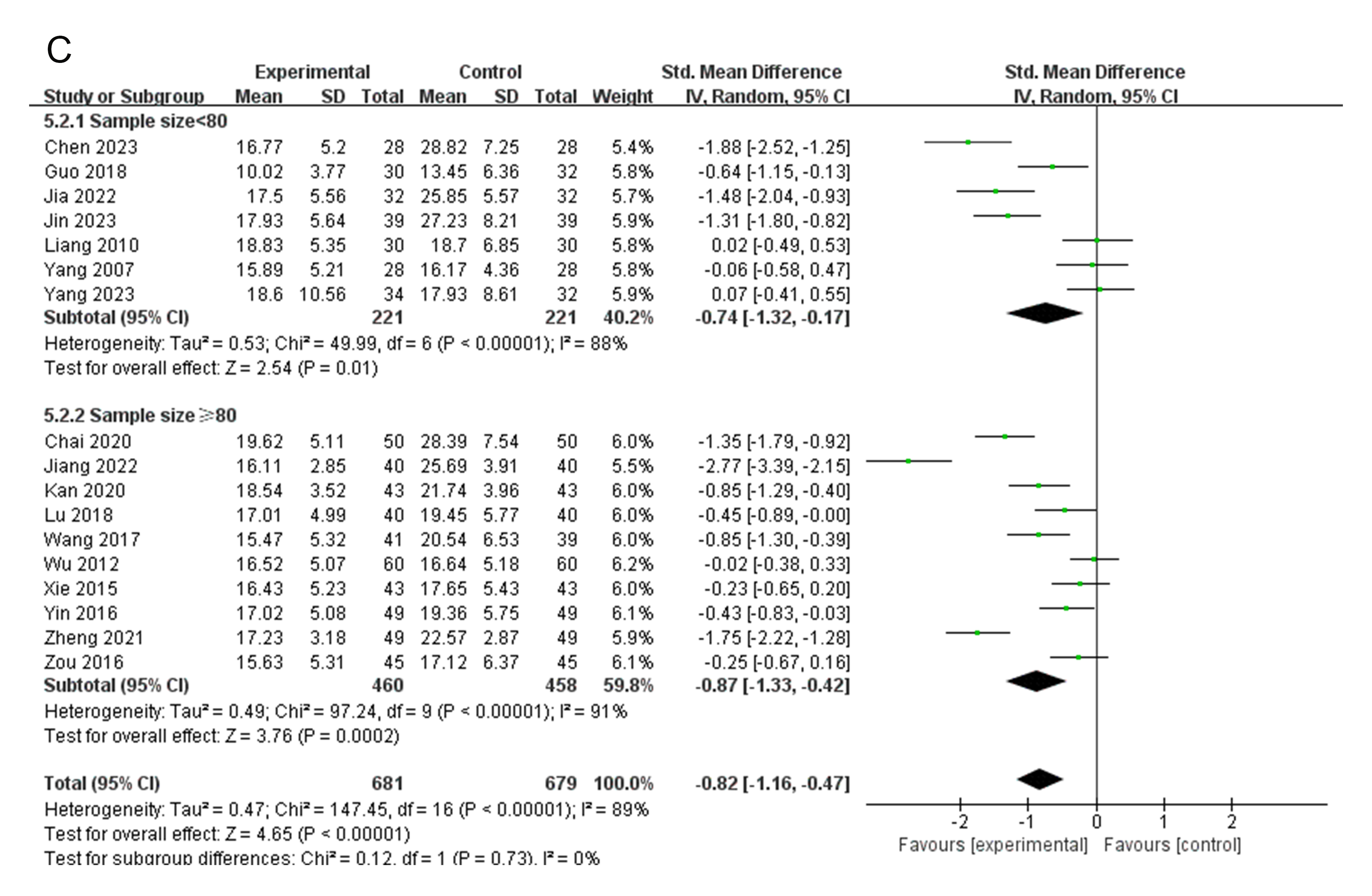


# **Supplementary Fig. 3 Subgroup analysis of TSH**

(A)Intervention duration (T < 6months, T ≥ 6 months); (B) PVL preparations (oral liquid, capsule, granule) (C) Sample size (sample size < 80, sample size ≥ 80).


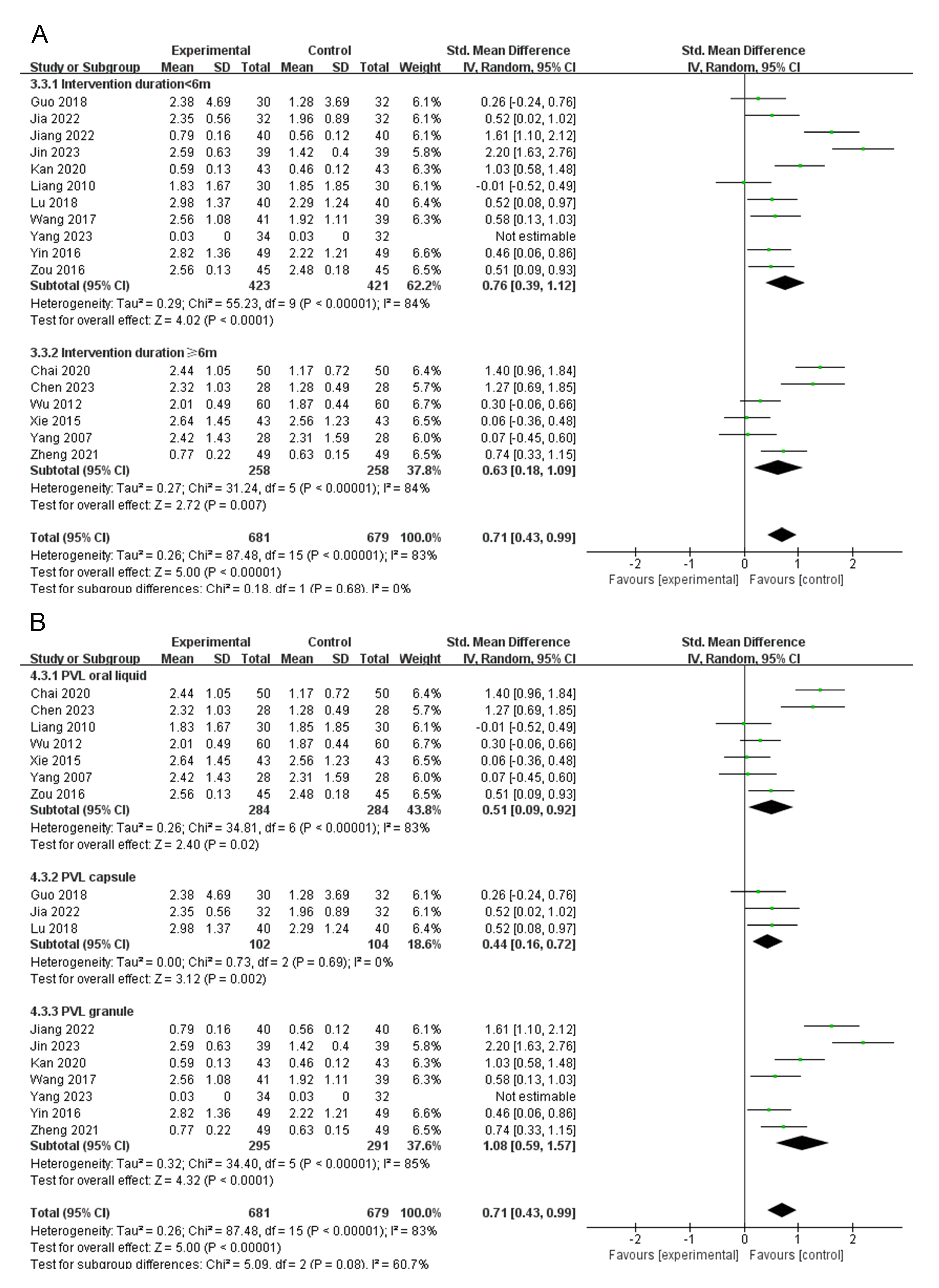


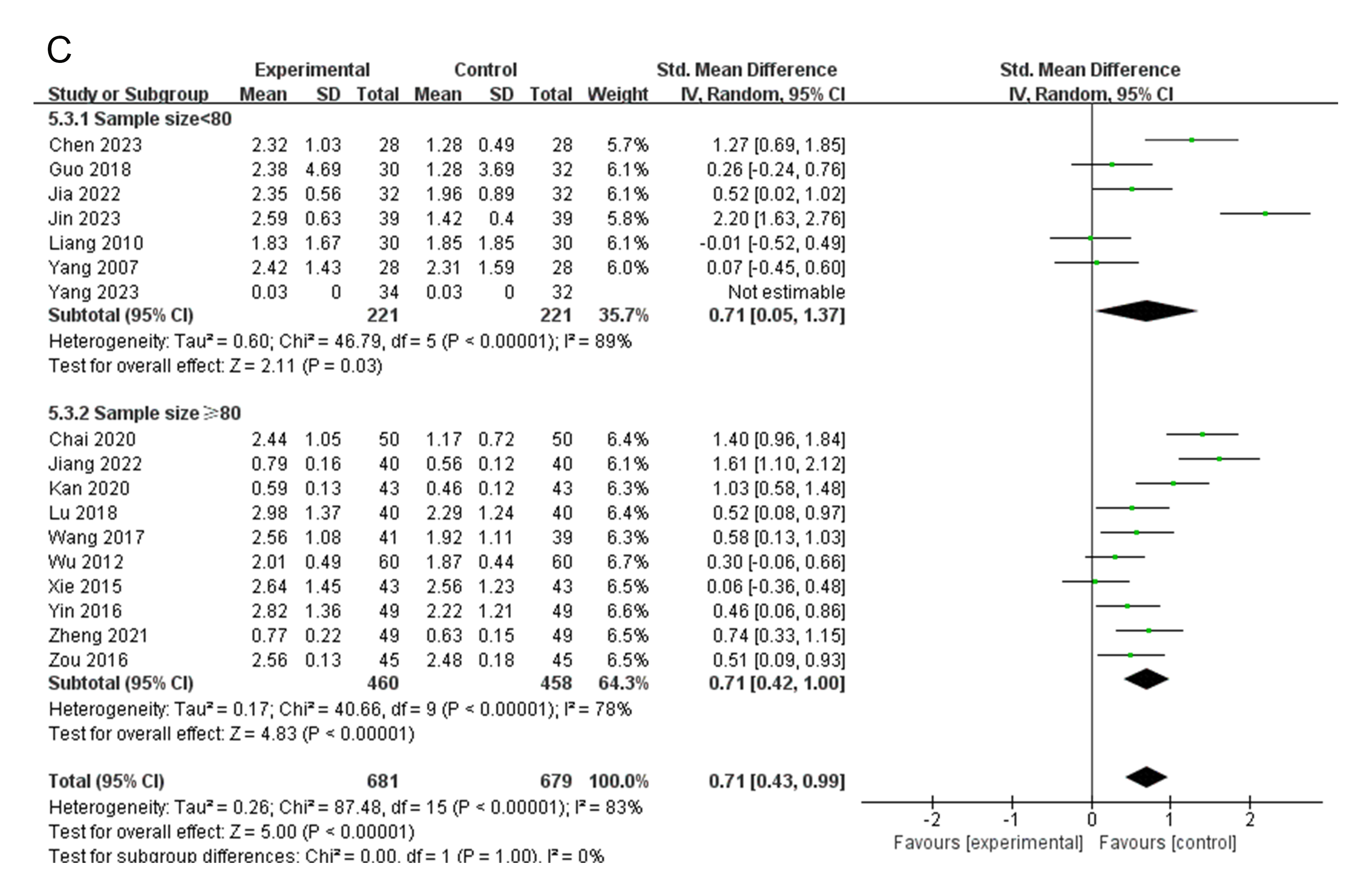


# **Supplementary Fig. 4 Subgroup analysis of TRAb**

(A) Intervention duration (T < 6months, T ≥ 6 months); (B) PVL preparations (oral liquid, capsule, granule) (C) Sample size (sample size < 80, sample size ≥ 80).


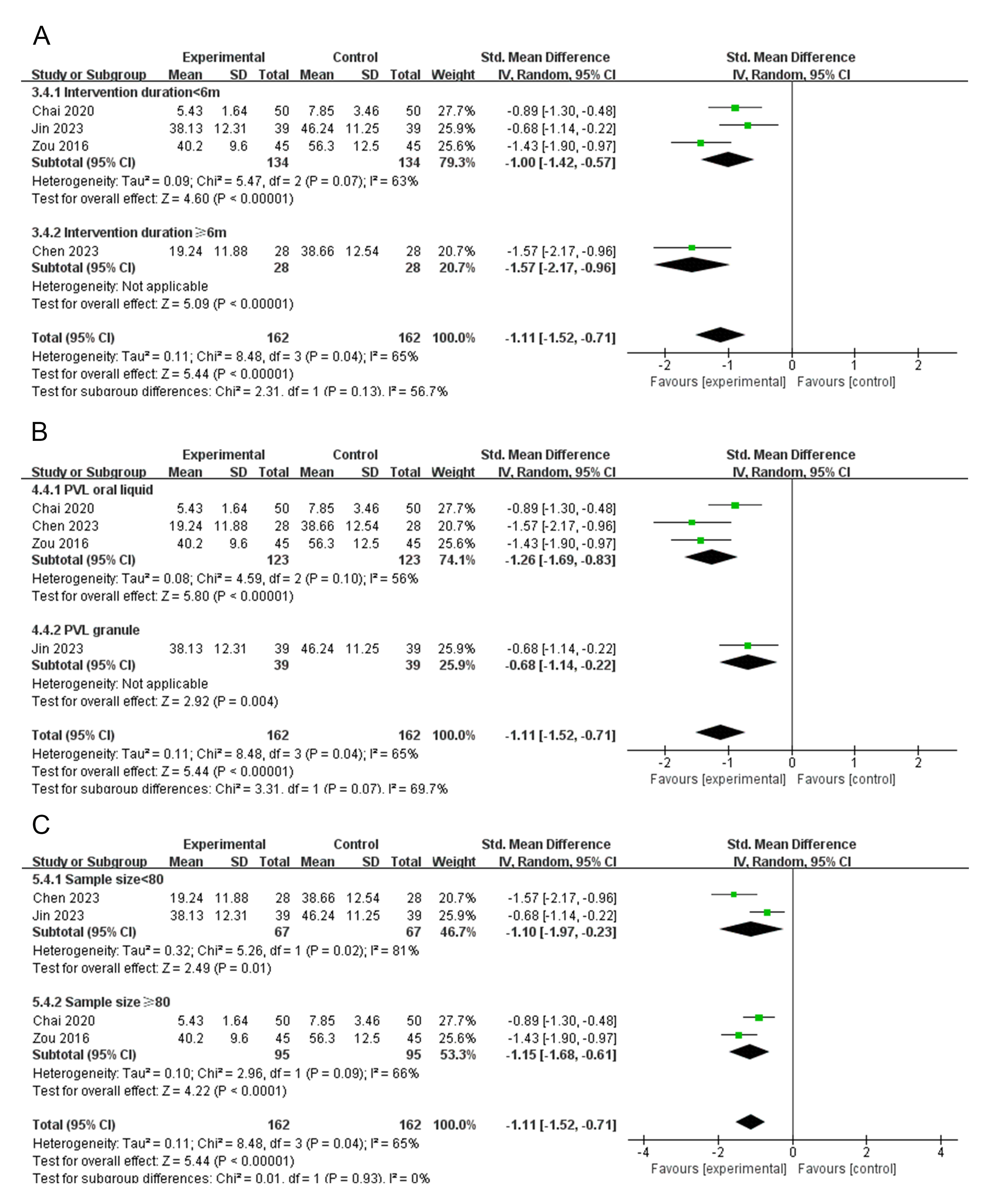


# **Supplementary Fig. 5 Subgroup analysis of TLTL**

(A) Intervention duration (T < 6months, T ≥ 6 months); (B) Sample size (sample size < 80, sample size ≥ 80).


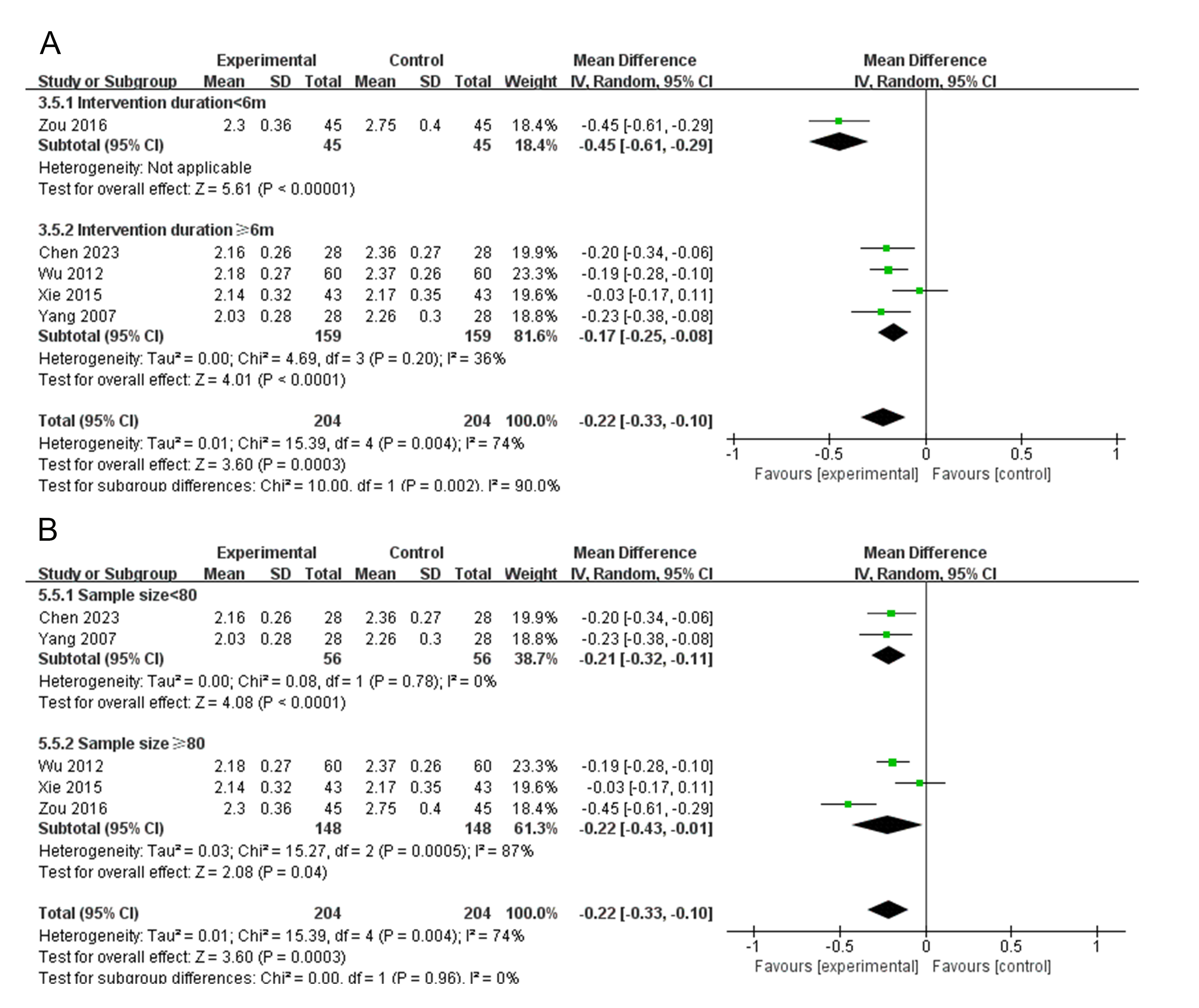


# **Supplementary Fig. 6 Subgroup analysis of** TNF-α and **IL-6**

(A) TNF-α (sample size < 80, sample size ≥ 80); (B) IL-6 (sample size < 80, sample size ≥ 80).


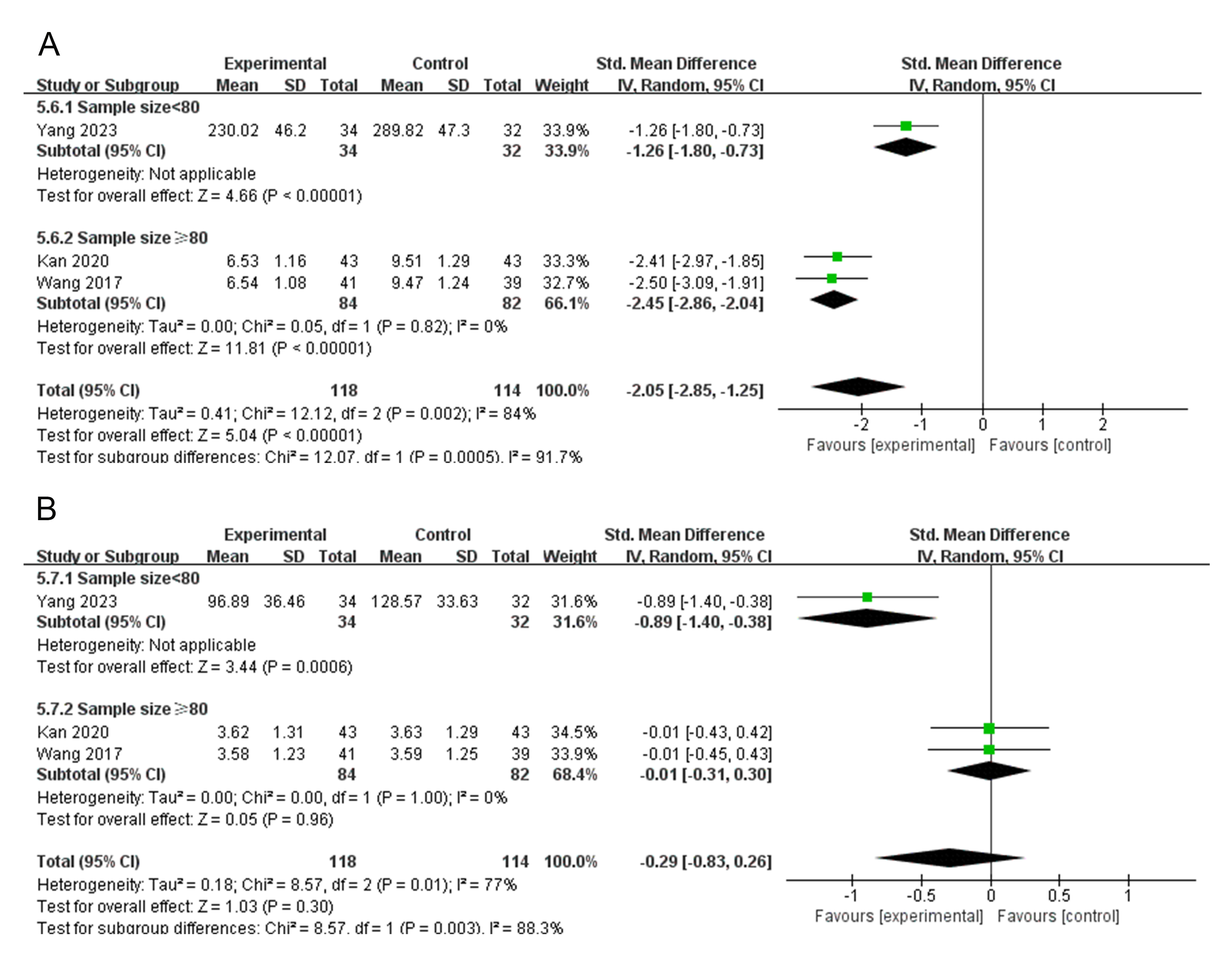

Supplement: Supplementary file 1 [file DataSheet1.docx]
